# Human Rev1 relies on insert-2 to promote selective binding and accurate replication of stabilized G-quadruplex motifs

**DOI:** 10.1093/nar/gkab041

**Published:** 2021-02-08

**Authors:** Amit Ketkar, Lane Smith, Callie Johnson, Alyssa Richey, Makayla Berry, Jessica H Hartman, Leena Maddukuri, Megan R Reed, Julie E C Gunderson, Justin W C Leung, Robert L Eoff

**Affiliations:** Department of Biochemistry and Molecular Biology, University of Arkansas for Medical Sciences, Little Rock, AR 72205, USA; Department of Biochemistry and Molecular Biology, University of Arkansas for Medical Sciences, Little Rock, AR 72205, USA; Arkansas School for Mathematics, Sciences, and the Arts, Hot Springs, AR 71901, USA; Arkansas School for Mathematics, Sciences, and the Arts, Hot Springs, AR 71901, USA; Department of Biochemistry and Molecular Biology, University of Arkansas for Medical Sciences, Little Rock, AR 72205, USA; Department of Biochemistry and Molecular Biology, Medical University of South Carolina, Charleston, SC 29425, USA; Department of Biochemistry and Molecular Biology, University of Arkansas for Medical Sciences, Little Rock, AR 72205, USA; Department of Biochemistry and Molecular Biology, University of Arkansas for Medical Sciences, Little Rock, AR 72205, USA; Department of Physics, Hendrix College, Conway, AR 72032, USA; Department of Radiation Oncology, University of Arkansas for Medical Sciences, Little Rock, AR 72205, USA; Department of Biochemistry and Molecular Biology, University of Arkansas for Medical Sciences, Little Rock, AR 72205, USA

## Abstract

We previously reported that human Rev1 (hRev1) bound to a parallel-stranded G-quadruplex (G4) from the *c-MYC* promoter with high affinity. We have extended those results to include other G4 motifs, finding that hRev1 exhibited stronger affinity for parallel-stranded G4 than either anti-parallel or hybrid folds. Amino acids in the αE helix of insert-2 were identified as being important for G4 binding. Mutating E466 and Y470 to alanine selectively perturbed G4 binding affinity. The E466K mutant restored wild-type G4 binding properties. Using a forward mutagenesis assay, we discovered that loss of hRev1 increased G4 mutation frequency >200-fold compared to the control sequence. Base substitutions and deletions occurred around and within the G4 motif. Pyridostatin (PDS) exacerbated this effect, as the mutation frequency increased >700-fold over control and deletions upstream of the G4 site more than doubled. Mutagenic replication of G4 DNA (±PDS) was partially rescued by wild-type and E466K hRev1. The E466A or Y470A mutants failed to suppress the PDS-induced increase in G4 mutation frequency. These findings have implications for the role of insert-2, a motif conserved in vertebrates but not yeast or plants, in Rev1-mediated suppression of mutagenesis during G4 replication.

## INTRODUCTION

The landscape of the genome contains information beyond DNA sequence that governs biological systems, including the spatial arrangement of chromosomes, the composition and architecture of chromatin, and secondary structures adopted by certain sequence motifs. These epigenetic features regulate important functional responses for the cell. G-quadruplexes (G4) represent an important type of non-canonical DNA structure with ramifications for DNA replication programs and gene expression profiles, as well as post-transcriptional events. Sequence motifs capable of adopting G4 structures occur non-randomly throughout the genome of all organisms, often at key regions like replication origins, promoters, and telomeres (1). They are formed by tandem tracts of 2–4 guanine bases, such that four guanines interact through the non-conventional Hoogsteen hydrogen bonds to form a planar ring, sometimes referred to as a G-tetrad or G-quartet (2). G-quartets stack on top of each other, associating via inter-planar pi bonds, to stabilize the four-stranded quadruplex structure. Further stabilization is achieved by the coordination of monovalent cations like Na^+^ or K^+^ with the O6 atoms in the central channel of the G-quartet. G4-DNA structures, once formed, can be very stable *in vitro*, often with significantly higher melting temperatures as compared to B-form DNA (3).

The existence of G4 structures in cells and *in vivo* is supported by orthogonal studies in a variety of organisms (4). Maximally formed during S-phase of the cell cycle, the four-stranded secondary structure of G4 DNA represents an endogenous barrier to the replisome (5). Consequently, the coordinated interplay of multiple proteins equipped to resolve these structures is required for effective G4 maintenance. Many studies have investigated the G4 unwinding by helicases, such as Pif1, FANCJ, BLM, and WRN (among others)—revealing key features important for G4-selective activity (2). The consequence of defective G4 unwinding is apparent from the increased genomic instability and epigenetic defects observed when the action of these helicases is lost. Additional studies have implicated other DNA replication and repair factors, such as the repair polymerase pol θ and translesion DNA synthesis polymerases (TLS pols), in the suppression of genomic and epigenomic instability near G4 motifs (6,7). The exact nature of TLS enzyme participation in G4 replication remains unclear, but a number of studies have implicated the Y-family member, Rev1, as a central player in effecting the bypass of G4 motifs.

As part of the replication stress response, Rev1 helps promote continuous synthesis past a variety of blocks to replication and in response to nucleotide depletion (8). The role of Rev1 in G4 replication is likely multifaceted. Rev1 is atypical among polymerases in that it uses a protein-template guided mechanism of nucleotide selection, which almost exclusively inserts dCMP and severely limits processive DNA synthesis. Avian cells lacking Rev1 exhibit deficiencies in G4 replication and maintenance of the epigenetic landscape surrounding quadruplex-forming sequences (9,10). Like tolerance of DNA adducts, the replisome adapts to endogenous replication blocks, and Rev1 is part of a complex of proteins that promotes G4 bypass. A key role for Rev1 is related to a motif in the C-terminal domain that aids in the recruitment of multiple TLS pols to sites of replication stress (11–14). Deletion of the Rev1 C-terminus reduced the efficiency of G4 bypass, with complete loss of Rev1 leading to defective histone cycling and loss of epigenetic regulation of gene expression. Both avian and human Rev1 (hRev1) could recover G4 replication efficiency in *rev1* mutant DT40 cells, but catalytically inactive Rev1 only partially restored the replication efficiency observed in wild-type cells (9). In addition to TLS pol recruitment, Rev1 functions in concert with the FANCJ helicase to promote replication across sequences prone to fold into quadruplex structures (15,16). These findings served as the rationale for biochemical studies on human Rev1 (hRev1) interactions with G4 DNA.

We previously found that hRev1 binds G4-DNA substrates with up to 15-fold greater affinity compared to non-G4 sequences (17). Further, hRev1 mechanically disrupted and prevented refolding of G4 structures *in vitro* through a mechanism that did not require nucleotidyl transfer (17). Yet, a number of questions remain unanswered concerning how hRev1 interacts with G4 DNA. Knowledge of the molecular interface between Rev1 and G4 would improve our understanding of features that may be important determinants of biological function during replication of structured DNA. As such, the biochemical mechanism and the ramifications for hRev1 action during G4 replication in human cells both remain active areas of investigation. In the present study, we used biochemical approaches to identify regions of Rev1 important for its interaction with G4 DNA and then validated the biological relevance of these regions to accurate replication of G4 motifs in human cells.

## MATERIALS AND METHODS

### Reagents and services

All chemicals and reagents used in this study were molecular biology grade, or better. The dNTP solutions were purchased from GE Healthcare Life Sciences (Piscataway, NJ, USA) and Thermo-Fisher Scientific (Waltham, MA, USA). All synthetic oligonucleotides were purchased from Integrated DNA Technologies (Coralville, IA, USA) desalted and validated by mass spectrometry. *p*-Hydroxyphenylglyoxal (HPG) was purchased from ProteoChem (Hurricane, UT, USA). The HAP-1 cell lines (parental hRev1-proficient and *REV1*^KO^) were obtained from Horizon Discovery (Cambridge, UK), while the HEK293T cells was obtained from American Type Culture Collection (ATCC; Manassas, VA, USA). Knock-out of the *REV1* gene was confirmed by the company using Sanger sequencing. We confirmed deletion of hRev1 protein expression by immunoblotting (data not shown). A mouse monoclonal anti-Rev1 antibody was used (Santa Cruz Biotechnology, Dallas, TX, USA; Catalog #sc-393022). DNA sequencing to confirm mutations in plasmids used for expression of recombinant hRev1 (wild-type and mutants) and to determine the mutagenic profile of the samples from the *supF* forward mutagenic assay was performed at the DNA Sequencing Core facility at UAMS. The tryptic digestion of HPG-reacted hRev1 and subsequent analysis of the HPG-labeled samples by mass spectrometry was performed at the UAMS Proteomics Core facility.

### DNA substrate preparation

Oligonucleotides were resuspended in 50 mM HEPES buffer (pH 7.5). Substrates for the fluorescence polarization experiments were prepared by annealing each of the 5′-FAM-labeled G4 and non-G4 oligos shown in Table 1 with the 11-mer primer (1:2 molar ratio of template:primer) in a 50 mM HEPES buffer (pH 7.5) containing 100 mM KCl/LiCl. For ssDNA substrates, the 11-mer primer was excluded. The mixture was heated at 95°C for 5 min, followed by slow-cooling to room temperature. Circular dichroism (CD) spectroscopy was performed to validate potassium-dependent G4 formation (Supplementary Figure S1). The annealed substrates were stored at room temperature in amber-colored tubes in the dark. The primer-template substrates for enzyme activity assays were prepared similarly by mixing the 42-mer G4 or non-G4 template oligonucleotides with the 5′-FAM-labeled 23-mer primer (1.5:1 molar ratio of template:primer) in 40 mM HEPES buffer (pH 7.5) containing 40 mM KCl. These were then annealed by heating to 95°C for 5 min, followed by slow-cooling to room temperature. The G4 and non-G4 oligos were prepared for the circular dichroism studies by resuspending them in 10 mM Tris–HCl buffer (pH 7.5) containing 100 mM KCl/LiCl. The substrates were heated to 95°C for 5 min, followed by slow-cooling to room temperature to allow the G-quadruplexes to form. Substrates were stored at room temperature.

**Table 1.** Sequences of G4- and nonG4-DNA substrates used in this study^a^

Name	Sequence	Type of G4 fold
Myc 14/23	5′-FAM-A**GGG**T**GGG**TA**GGG**T**GGG**T*TATGAGATGAT* -3′	Parallel
Myc 2/11	5′-FAM-A**GGG**A**GGG**T**GGG**AA**GGG**T*TATGAGATGAT* -3′	Parallel
Non-G4 Myc control	5′-FAM-AGCGTGCGTAGCGTGCGT*TATGAGATGAT* -3′	-
KRAS 22RT	5′-FAM-A**GGG**C**GG**T**G**T**GGG**AAGA**GGG**AA*TATGAGATGAT* -3′	Parallel
Non-G4 KRAS control	5′-FAM-AGCGCGCTGTGCGAAGAGCGAA*TATGAGATGAT* -3′	-
Bcl2 1245	5′-FAM-A**GGGG**C**GGG**CGCTTTAGGAAAA**GGG**C**GGG**T*TATGAGATGAT*-3′	Parallel
Non-G4 Bcl2 control	5′-FAM-AGCGGCGCGCGCTTTAGGAAAAGCGCGCGT*TATGAGATGAT*-3′	-
Rev1-prom	5′-FAM-A**GGG**C**GGG**GCC**GGG**GA**GGG***TATGAGATGAT* -3′	Parallel
Non-G4 Rev1-prom control	5′-FAM-AGCGCGCGGCCGGCGAGCG*TATGAGATGAT* -3′	-
TBA	5′-FAM-T**GG**TT**GG**TGT**GG**TT**GG***TATGAGATGAT* -3′	Antiparallel
Non-G4 TBA control	5′-FAM-TGCTTGCTGTGCTTGC*TATGAGATGAT* -3′	-
hTelo-4	5′-FAM-TT**GGG**TTA**GGG**TTA**GGG**TTA**GGG**A*TATGAGATGAT* -3′	Hybrid
Non-G4 Telo-4 control	5′-FAM-TTGCGTTAGCGTTAGCGTTAGCGA*TATGAGATGAT* -3′	-
11-mer primer	5′- ATCATCTCATA -3′	-
42-mer G4-template^b^	5′-TGA**GGG**T**GGG**TA**GGG**T**GGG***TGCGTCTGCGGCTGGCTCGAGGC*-3′	Parallel
42-mer non G4-template^b^	5′-GTGAGATGTTGACCATGGG*TGCGTCTGCGGCTGGCTCGAGGC*-3′	-
23-mer 5′ FAM-primer^b^	5′-FAM-TTTGCCTCGAGCCAGCCGCAGACGCA-3′	-

^a^The guanine bases involved in the formation of G4-tetrads are shown in bold. The corresponding non-G4 oligonucleotides had C in place of a G (underlined in each sequence). A single non-G4 Myc control sequence was used for both the Myc-14/23, and Myc-2/11 G4 sequences. Shown in italics is the region of each oligonucleotide that is complementary to the common 11-mer primer. The 5′-FAM-labeled versions of all substrates shown above were used in the fluorescence polarization experiments.

^b^The 42-mer template and 23-mer primer sequences were used exclusively in assays to determine polymerase activity. Shown in italics in the region of each oligonucleotide that is complementary to the 23-mer primer.

### Generation of mutant hRev1 proteins and purification

Expression and purification of hRev1^330–833^ (the polymerase core) from *Escherichia coli* was performed as previously described (17). Mutations in the hRev1^330–833^ construct were generated by site-directed mutagenesis, using the following primers: (a) E466A: forward primer – 5′-CCCAGCTGGCGTGGCAGTATTAC-3′; reverse primer – 5′-GTAATACTGCCACGCCAGCTGGG-3′. (b) E466K: forward primer – 5′-CCCCAGCTGAAATGGCAGTATTAC-3′; reverse primer – 5′-GTAATACTGCCATTTCAGCTGGGG-3′. (c) W467A: forward primer – 5′-CCCAGCTGGAGGCGCAGTATTACC-3′; reverse primer – 5′-GGTAATACTGCGCCTCCAGCTGGG-3′. (d) Y470A: forward primer 5′-GGCAGTATGCCCAGAATAAAATC-3′; reverse primer – 5′-GATTTTATTCTGGGCATACTGCC-3′. (e) L358A: forward primer – 5′-CTATTCTCATTCAAGAGCGCATCACATATC-3′; reverse primer – 5′-GATATGTGATGCGCTCTTGAATGAGAATAG-3′. Mutant enzymes were expressed and purified using the same protocol as wild-type enzyme (Supplementary Figure S2). Full-length hRev1^1–1251^ was cloned into the p-BABE vector using the Gateway™ cloning system (Thermo-Fisher; Waltham MA, USA) to express hRev1 as a N-terminal S-protein-FLAG-Biotin (SFB)-tandem affinity tagged protein (Supplementary Figure S3). For expression and purification, HEK293T cells stably expressing the SFB-tagged hRev1^1–1251^ protein were generated. These cells were then cultured on a large scale in five to ten 175 cm^2^ tissue culture flasks in Dulbecco's modified Eagle medium supplemented with 10% (v/v) fetal bovine serum and 1% (v/v) antibiotics for a final concentration of 100 units/mL penicillin, 100 μg/ml streptomycin, and 0.25 μg/ml amphotericin B (Sigma-Aldrich, St. Louis, MO, USA). Cells were cultured in an incubator at 37°C in an atmosphere containing 5% (v/v) CO_2_. Cultures were harvested at >80% confluency (∼15–20 × 10^6^ cells per flask), and the cell pellet was lysed in a 40 mM HEPES buffer (pH 7.5) containing 120 mM NaCl, 1 mM EDTA, 1% (v/v) Triton-X 100, and protease and phosphatase inhibitors. The clear lysate obtained after centrifugation was incubated with streptavidin–Sepharose beads (GE Healthcare) at 4°C for 16 h to enable the SFB-hRev1 protein to bind. After washing the beads thoroughly to remove non-specifically bound proteins, the SFB-hRev1 was eluted in a buffer containing 2 mg/ml biotin. The eluate was dialysed to remove biotin, followed by concentration of the eluted protein using spin concentrators (Amicon). Purity of eluted hRev1^1–1251^ protein was confirmed by SDS-PAGE followed by Ponceau staining, as well as immunoblotting using an anti-FLAG-tag antibody (Supplementary Figure S3; Novus Biologicals, Centennial, CO, USA).

### DNA binding by fluorescence polarization

The affinity of the purified hRev1^330–833^ wild-type and mutant proteins, as well as the purified hRev1^1–1251^ protein towards all the 5′-FAM labeled non-G4 and G4 DNA substrates described in this study, was determined using fluorescence polarization on a plate-reader (Biotek SynergyH4), as described previously (17). Briefly, titrations of various concentrations of each protein with a fixed concentration (1 nM) of all the DNA substrates were performed in buffer containing either 100 mM KCl or 100 mM LiCl. The change in fluorescence polarization at every titration was measured, and plotted as a function of protein concentration, and fitted to a quadratic equation as described previously (17), to determine the values of equilibrium dissociation constants.

### Measurement of enzyme activity

In order to determine the effect of mutations on the catalytic efficiency of hRev1^330–833^, the enzymatic activity of all the mutant hRev1 proteins was determined with non-G4 and G4 DNA template–primer substrates and compared to the activity of the wild-type protein. All assays were performed in 40 mM HEPES buffer (pH 7.5) containing 100 mM KCl, 0.1 mg/mL BSA, and 5 mM dithiothreitol at 37°C using 50 nM protein and 200 nM DNA. Reactions were initiated by adding a mixture of 5 mM MgCl_2_ and 1 mM dCTP. At intervals of 0, 10, 20, 40, 60 and 90 min, 15 μl aliquots were withdrawn from each reaction mixture and added to tubes containing 85 μl of quench solution (95% v/v formamide, 20 mM EDTA, 0.1% w/v bromophenol blue), and heated at 95°C for 5 min. Aliquots of these quenched reactions were loaded on to 7 M urea/14% (w/v) acrylamide gels and electrophoresed at a constant power of 35 W for 2–3 h to separate the products. Gels were scanned on a Typhoon Imager (GE Life Sciences, Marlborough, MA, USA), and the bands corresponding to substrate and products in each lane were quantified using the ImageJ software (18). Total product formation for each protein with both the DNA substrates was plotted as a function of time. This was converted to percent product formed per unit time, by dividing the sum of intensities of all product bands in a lane by the total sum of intensities of all bands in that lane. The initial portion of the velocity curve was fit to a linear equation to estimate the rate of product formation. Activities were normalized to the activity of wild-type enzyme (taken as 100% with non-G4 DNA) for both DNA substrates. Relative activity of each protein was also calculated for the non-G4 and G4 substrates by dividing their respective percent product values.

### Chemical footprinting of hRev1 by HPG-modification of arginines

Chemical labelling of hRev1^1–1251^ was performed using the arginine-reactive chemical HPG, as described previously (19). Briefly, 10 μM of purified hRev1^1–1251^ protein was allowed to react with 0.5 mM HPG in 50 mM HEPES (pH 7.5) buffer containing 100 mM KCl. Prior to addition of HPG, the protein was pre-incubated with either buffer (no DNA/control), 30 μM of single-stranded (ss)-Myc G4, ss-TBA G4 or ss-non G4 DNA for 15 min prior to HPG modification. All the ssDNA substrates were prepared by heating them to 95°C for 5 min in 25 mM HEPES buffer (pH 7.5) containing 100 mM KCl, and then cooling them overnight to room temperature. One hour after incubating with HPG (in the dark) at room temperature, the reactions were quenched by the addition of 200 mM l-arginine. Laemmli buffer (1×) was added to each sample, and it was heated to 95°C for 5 min before loading on a 4–20% SDS-PAGE gradient gel for electrophoresis. The gel was stained by Coomassie Blue stain, and the band for hRev1^1–1251^ protein in each lane was excised and submitted to the UAMS Proteomics Core for tryptic digestion and mass spectrometric analysis. Experiments were performed in duplicate. Results from the mass spectrometric analysis were inspected using the Scaffold Viewer software (Proteome Software Inc.). The unmodified and HPG-modified peptides were identified for each sample. The peak intensity for both the unmodified and HPG-modified version of a peptide was determined manually by calculating the area under the curve from the total ion chromatogram for the parent ion using the XCalibur software (Thermo Fisher Scientific). The extent of HPG-modification at every arginine identified by mass spectrometry was calculated as a ratio of the HPG-modified intensity to the total intensity value for that arginine (unmodified + modified). Results were normalized across samples, by considering the HPG-modification in the no-DNA sample as 100%.

### *supF* forward mutagenesis assay

We engineered an 18 nucleotide G4 forming sequence derived from the c-Myc promoter immediately upstream of the *supF* tRNA coding region in the pSP189 plasmid (Supplementary Figure S4). The original pSP189 plasmid served as a non-G4 control (20). Cloning of the G4 fragment into psP189 was performed using three sequential PCR reactions to insert the 18 nt sequence in three parts, using the following primer pairs: PCR reaction 1, forward primer 5′-GAGCCCTGCTCGAGCTGTGGAGGGTGGGGTTCCCGAGCGG-3′, reverse primer 5′-CCGCTCGGGAACCCCACCCTCCACAGCTCGAGCAGGGCTC-3′. PCR reaction 2, forward primer 5′-GAGCCCTGCTCGAGCTGTGAGGGTGGGGAGGGTGGGGTTCC-3′, reverse primer 5′-GGAACCCCACCCTCCCCACCCTCACAGCTCGAGCAGGGCTC-3′. PCR reaction 3, forward primer 5′-GAGCCCTGCTCGAGCTGTGGGGAGGGTGGGGAGGGTGGG-3′, reverse primer 5′- CCCACCCTCCCCACCCTCCCCACAGCTCGAGCAGGGCTC-3′. After each PCR step, the resulting products were sequenced to confirm the insertion of a portion of the 18 nt G4 sequence. PCR product with the correct sequence served as the template for the next PCR reaction.

Both the plasmids were transfected into HAP-1 cells (Horizon Discovery, Cambridge, UK), which either expressed wild-type hRev1 (HAP-1) or had the *hRev1* gene knocked out by CRISPR-Cas9 (*REV1^KO^*). The HAP-1 cells were cultured in Iscove's modified Dulbecco's medium (Millipore-Sigma, St. Louis, MO, USA) supplemented with 10% (v/v) fetal bovine serium and 1% (v/v) antibiotics containing 100 units/mL penicillin, 100 μg/ml streptomycin, and 0.25 μg/ml amphotericin B (Sigma-Aldrich, St. Louis, MO, USA). Cells were cultured in an incubator at 37°C in an atmosphere containing 5% (v/v) CO_2_. Transfection was performed using Lipofectamine-3000 (Thermo Fisher, USA) according to the manufacturer's instructions. Ectopic expression of hRev1 was confirmed by immunoblotting (Supplementary Figure S5). The transfected cells were cultured either in the presence or absence of the G4-stabilizing chemical pyridostatin (PDS; 0.5 μM) for 24 h and then harvested. The pSP189 plasmids (control or G4) were extracted from cell pellets using the plasmid miniprep kit (Qiagen). Following treatment with the Dpn I endonuclease to digest parental (methylated) DNA, the plasmids were then used to transform the MBM7070 indicator strain of *E. coli*, and transformants were plated on LB-agar plates containing 100 μg/ml ampicillin, 2 mg/ml 5-bromo-4-chloro-3-indolyl-β-d-galactopyranoside (X-Gal) and 1 mM isopropyl-β-d-1-thiogalactopyranoside (IPTG). After incubation at 37°C for 15 h, blue and white colonies were observed on the plates. If necessary, the plates were incubated overnight at 4°C to allow the blue colour to intensify. The number of blue (unmutated) and white (mutated) colonies were counted for each plate using the Fiji version of ImageJ software. Mutation frequency was determined for each experimental sample by plotting the ratio of number of white colonies for a sample, to the total number of colonies (blue and white) for that sample. Each experimental condition was performed in triplicate. For every replicate, at least 10 000 colonies were counted. Further, to investigate the mutagenic profile of the *supF* gene vicinity region, plasmids from representative white colonies were extracted and submitted for DNA sequencing. For every experimental condition, 10 white colonies were sequenced (Supplementary Figures S6 and S7). The mutant sequences were then aligned using the T-Coffee alignment server (21). The region in the vicinity of the *supF* gene was divided into four zones. The nature and frequency of occurrence of mutations was determined by examination of every zone across all the samples. Mutations identified by sequencing were either base substitutions (transversions and transitions) or deletions. We divided the sequence surrounding the *supF* gene into four zones: zone 1—containing nts on the 5′ side of the G4 motif, zone 2—including the G4 motif, zone 3—including the *supF* gene and zone 4—including nts on the 3′-side of the *supF* gene. The mutation profile was calculated by counting the number of base substitutions and deletions for a given number of nucleotides in each zone. Statistical analysis was performed using Graphpad Prism (San Diego, CA, USA).

## RESULTS

### Substrate design and characterization

We have shown that hRev1^330–833^ binds G4 DNA with greater affinity than non-G4 substrates (17). Our earlier study used a G4-forming sequence that was based on a motif found in the *c-MYC* promoter (Table 1, Myc 14/23). The nomenclature for the Myc G4 substrates is related to changes to the wild type purine-rich 27mer G4 motif where multiple runs of three (or more) guanines exist in tandem. Mutating the 14th and 23rd guanines to thymidine prevents formation of multiple G4 structures and improves the uniformity of the G4 folds formed *in vitro* (22). Similarly, the Myc 2/11 substrate was designed by mutating residues 2 and 11 from the wild type G4 motif. The Myc 14/23 sequence was originally selected because it is an extensively characterized G4 motif that is known to form a stable and relatively uniform parallel-stranded quadruplex structure (23,24). We wanted to investigate whether preferential binding to G4 DNA by hRev1 extended to other G4 motifs and folds. We also wanted to test whether changing the stability, loop-length, or inserting a base between tandem guanines would change the preference of hRev1 for binding to a parallel-stranded G4 substrate. To achieve this objective, we designed a panel of G4 substrates (Table 1). A non-G4 control sequence of the same length and with similar GC content was designed for every G4 substrate.

CD spectroscopy was used to validate the formation of G4 structure in 100 mM KCl buffer for all substrates (Supplementary materials and methods). A characteristic positive peak at ∼264 nm and a negative peak at ∼240 nm was observed for the parallel-stranded G4 substrates (Supplementary Figure S1). The melting temperature (*T*_m_) for each G4 structure was also calculated for all substrates by measuring change in CD signal as a function of temperature (Supplementary Table S1). For DNA binding assays, five parallel-stranded G4 substrates were prepared. Two *c-MYC*-derived sequences were included, Myc 14/23 and Myc 2/11. These two sequences both form parallel-stranded G4 structures but they differ in loop arrangement, with the more stable Myc 14/23 possessing a 1:2:1 loop arrangement between runs of guanine and Myc 2/11 possessing a 1:1:2 loop arrangement between guanines (Table 1). The KRAS 22RT derived substrate folds into a parallel-stranded quadruplex as well, but the sequence includes a 1:1:4 loop arrangement with single thymidine interrupting the second run of three guanines. The nomenclature for the KRAS G4 structure stems from the original motif being named 32R (25). Truncation of the 32 nt sequence to a core 22 nucleotides along with mutation of a guanine to thymidine (22RT) stabilized the parallel-stranded structure and prevented alternative G4 folds (26). The Bcl2 G4 motif in the P1 promoter of the *BCL-2* gene controls expression of this anti-apoptotic protein (27). The wild type Bcl2 G4 motif is 39 nucleotides long and includes six runs of guanine (28). While 15 different folds are possible, two major G4 species are thought to arise from the wild type 39mer sequence: Bcl2 1245 and Bcl2 2345, where the numbers represent the involvement of the different runs of guanine. For the Bcl2 1245 sequence, the third run of guanines was mutated to three thymidines to improve the uniformity of the G4 structure *in vitro* (28). The Bcl2 1245 G4 motif folds into a parallel stranded G4 but this sequence possesses a 13 nt loop between the second and third guanine runs. Finally, we used the QGRS algorithm to identify multiple putative G4 motifs in the *REV1* promoter (Supplementary Figure S8). The highest scoring motif was found >1760 bp upstream of the transcription start site. This site was identified previously as one of many *bone fide* G4 forming sequences located in the promoters of DNA repair and damage tolerance genes (29,30). We designed a substrate based on the top-scoring motif (Rev1-prom) and evaluated it for G4-related properties. The Rev1-prom G4 motif is comprised of 18 nts, including four runs of three or more guanines. It has a 1:4:1 loop arrangement between guanines and appeared to form a parallel-stranded G4 structure based on CD analysis (Supplementary Figure S8). All the parallel-stranded G4 substrates were thermodynamically stable, based on their calculated melting temperatures (*T*_m_ between 56–90°C: Supplementary Table S1). As expected, when 100 mM LiCl was used instead of KCl, the stability of all G4 substrates was significantly reduced (as seen by much lower melting temperatures; Supplementary Table S1).

In addition to the parallel-stranded G4 substrates, we prepared a four-repeat human telomeric sequence (hTelo-4) to study hRev1 interactions with a hybrid G4 structure, while the thrombin binding aptamer (TBA) represented a relatively well-defined and stable anti-parallel G4 fold (Table 1). We confirmed using CD that the hTelo-4 oligonucleotide formed a mixed/hybrid G4 structure in 100 mM KCl, as has been previously reported (31). Further, we also confirmed that the TBA oligonucleotide sequence formed an anti-parallel stranded G4 structure in KCl buffer, with a positive peak at 290 nm and a negative peak at 260 nm (Supplementary Figure S1), as has been reported previously (32). The corresponding non-G4 control substrates of all sequences (non-G4 DNA) were tested similarly using CD to confirm that none of them formed quadruplex structures (data not shown).

### hRev1 exhibited preferential binding to parallel-stranded G4 DNA compared to anti-parallel and hybrid-type of G4 DNA substrates

**Figure 1.**
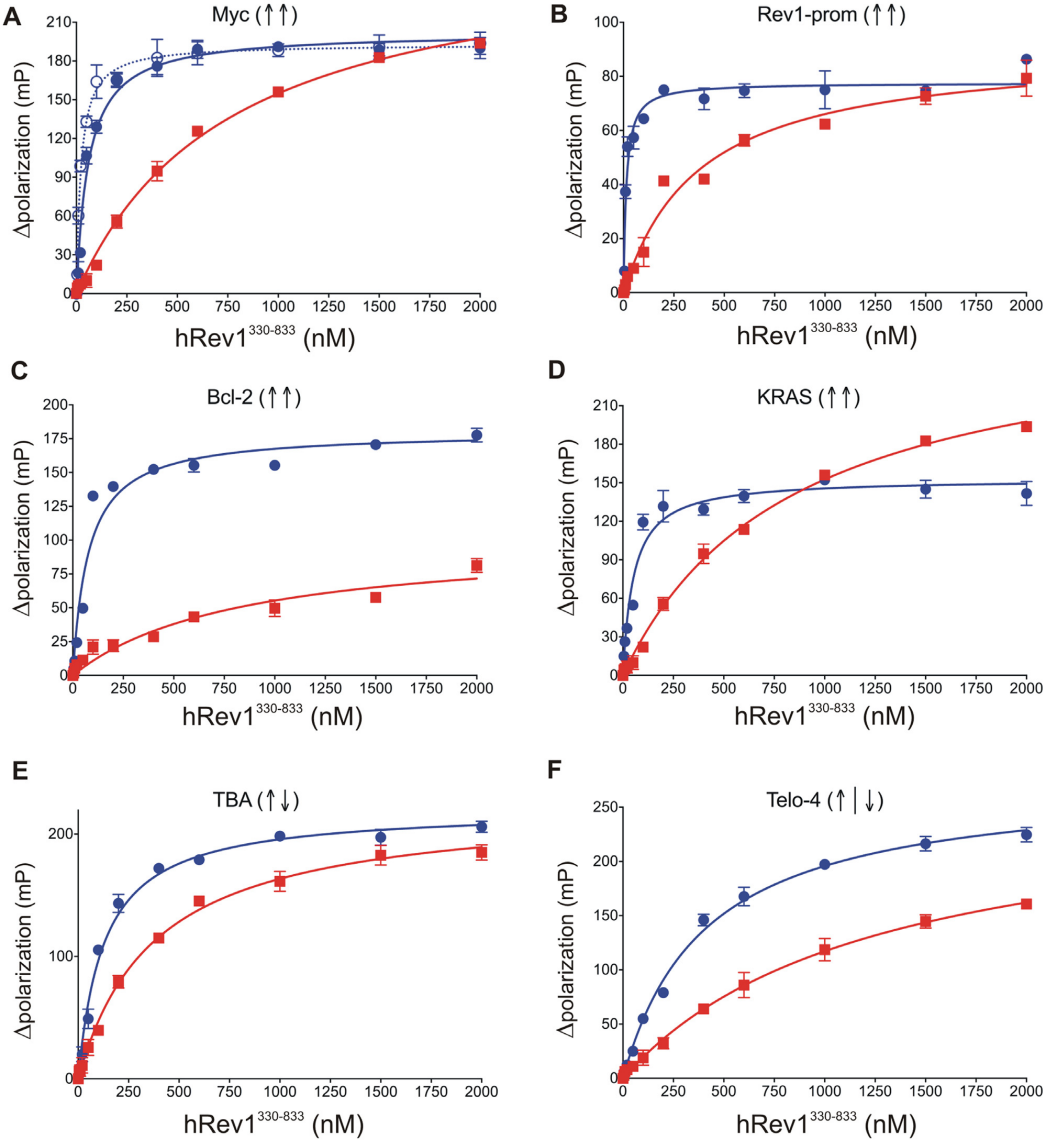
hRev1^330–833^ preferentially binds to G4-forming DNA sequences, with a greater affinity for parallel-stranded G4 DNA than other G4 folds. The hRev1^330–833^ protein was titrated into a solution containing either single-stranded (ss)-G4-DNA (*blue*) or ss-non-G4-DNA (*red*) substrates at 1 nM. The range of concentrations for the protein is indicated on the X-axis. The change in fluorescence polarization at each concentration was measured and plotted as a function of the protein concentration. (**A-F**) Binding curves for hRev1^330–833^ core protein with the indicated G4 DNA substrate. In panel A, the binding curve for Myc-14/23 is shown as a solid *blue* line (full circles), while that for Myc-2/11 is shown as a dotted *blue* line (open circles). The G4 fold is indicated by the direction of arrows in parentheses for each panel (↑↑ = parallel G4, ↑↓ = anti-parallel G4, ↑|↓ = hybrid G4). Resulting data were fit to a quadratic equation to yield the binding dissociation constants given in Table 2. Reported values represent the mean ± SD (*n* = 3).

Fluorescence polarization was used to monitor hRev1 binding to 5′-FAM-labeled DNA for each of the different G4 and non-G4 ssDNA substrates. By varying the concentration of hRev1 in the reaction mixture, we measured the equilibrium dissociation constant (*K*_D,DNA_) for binary complex formation. The resulting titration curves were fit to a quadratic equation (Figure 1). The results of the DNA binding assays are further summarized in Table 2 (ssDNA) and Supplementary Table S3 (dsDNA).

**Table 2.** Equilibrium dissociation constants for hRev1 binding to ss-G4 and non-G4 DNA substrates^a^

	*K*_D.DNA_		Fold preference for G4 DNA
	Non-G4 (nM)	G4 (nM)	(*K*_D,NonG4 DNA_/*K*_D,G4 DNA_)
**hRev1 (a.a. 1–1251)**			
Myc 14/23	110 ± 20	5 ± 1	20
**hRev1 (a.a. 330–833)**			
Myc 14/23	910 ± 120	37 ± 7	25
Myc 2/11	-	22 ± 3	41
Rev1-prom	370 ± 70	13 ± 3	29
Bcl-2 1245	830 ± 310	76 ± 16	11
KRAS 22RT	840 ± 90	52 ±10	16
TBA	380 ± 40	130 ± 10	3
hTelo-4	1200 ± 200	410 ± 40	3

^a^Fluorescence polarization experiments were performed by titrating hRev1 (either full-length, a.a. 1–1251, or pol core, a.a. 330–833) into a solution containing the indicated ss-DNA substrate in a buffer containing 100 mM KCl. The resulting equilibrium dissociation constant values were calculated by fitting the resulting polarization values to a quadratic equation. Similar measurements were also performed in buffer containing 100 mM LiCl (the values obtained are shown in Supplementary Table S2). Data represent the mean ± SD (*n* = 3).

As observed in our previous study, hRev1 exhibited greater affinity for the Myc 14/23 G4 sequence than the non-G4 control (Figure 1 and Table 2). In this case, hRev1^330–833^ exhibited an equilibrium binding constant of 37 ± 7 nM for Myc-14/23 ss-G4 DNA in 100 mM KCl. The value for binding constant for the corresponding non-G4 substrate was measured to be 910 ± 120 nM in 100 mM KCl. Thus, hRev1^330–833^ bound with ∼25-fold higher affinity to G4 Myc-14/23 as compared to the non-G4 control. This is in agreement with our observations reported earlier (17). Similar to the Myc 14/23 G4 substrate, hRev1 binding to the Myc 2/11 G4 substrate was much tighter than the non-G4 control (Figure 1A and Table 2). The preference for the 14/23 and 2/11 G4 folds was also evident from the fact that binding affinity was ∼5–7-fold tighter in presence of KCl compared to buffer containing LiCl (Supplementary Table S2). Substituting potassium with lithium reduced the difference in the hRev1^330–833^ binding constants for the Myc 14/23 G4 substrate and the non-G4 control from 25-fold for potassium to around 3-fold for the same substrates in LiCl (Table 2 and Supplementary Table S2). For the Myc 2/11 substrate, the preference for G4 DNA was reduced from ∼40-fold in KCl to ∼8-fold in lithium (Table 2 and Supplementary Table S2). The preferential binding to the Myc 14/23 G4 DNA substrate was also observed for full-length hRev1^1–1251^ (Table 2). The 20-fold preference for binding to G4 DNA for the full-length enzyme is comparable to the 25-fold difference observed for the polymerase core domain (a.a. 330–833). These results are in agreement with the notion that hRev1 binds to stable, parallel-stranded G4 structures with greater affinity than non-structured DNA.

The *K*_D,DNA_ values measured for the other parallel-stranded G4 substrates demonstrated trends in binding affinity that were similar to the Myc G4 motif. Interrupting the tandem guanines with a single thymidine did not alter hRev1^330–833^ binding to a parallel-stranded G4, as the *K*_D,DNA_ value for binding to the KRAS 22RT G4 substrate was 16-fold lower than that measured for the non-G4 control sequence in KCl (Figure 1 and Table 2). Inserting a 13 nt loop between tandem guanines also appeared to have a minimal impact on G4 selectivity, given that hRev1^330–833^ bound to the Bcl-2 1245 substrate with 11-fold greater affinity than the non-G4 control (Figure 1 and Table 2). A large difference in binding affinity between G4 and non-G4 DNA substrates was observed with the parallel-stranded G4 sequence from the human Rev1 promoter (Rev1-prom). In buffer containing potassium, the *K*_D,DNA_ for hRev1^330–833^ binding to the Rev1-prom sequence was 13 ± 3 nM compared with a *K*_D,DNA_ of 370 ± 70 nM for the non-G4 control (Figure 1 and Table 2). When these G4 oligonucleotides were suspended in LiCl, the difference in binding affinity for G4 and non-G4 substrates was reduced considerably (Supplementary Table S2). Thus, it would seem that the preferential binding to parallel-stranded G4 DNA over non-G4 sequences is a property that is inherent to hRev1 and not restricted to *c-MYC*-derived G4 sequences.

The trends observed for hRev1 binding to the hTelo-4 substrate were quite different from those of the parallel-stranded G4 substrates. In the presence of 100 mM KCl buffer, conditions under which the hTelo-4 sequence was confirmed to form a mixed/hybrid G4 structure, the measured binding constant to hRev1^330–833^ was 406 ± 38 nM. This was ∼7-fold lower affinity than that measured for the Myc-14/23 G4 substrate. A *K*_D,DNA_ value of 1246 ± 196 nM was measured for hRev1^330–833^ binding to the non-G4 sequence of equivalent length to the hTelo-4 substrate, which is only ∼3-fold greater than the hTelo-4 G4 substrate. The affinity was reduced by 1.5-fold when KCl was replaced with LiCl (634 ± 105 nM), an indication that hRev1^330–833^ does not exhibit a strong preference for binding to the hybrid hTelo-4 G4 structure.

Similar to the hTelo-4 results, hRev1^330–833^ bound to the anti-parallel G4 DNA substrate TBA with a *K*_D,DNA_ value of 127 ± 14 nM in 100 mM KCl, which is ∼2-fold higher than that measured for the Myc-14/23 G4 DNA. The observed value of dissociation constant for the non-G4 control was 380 ± 40 nM. The ∼3-fold preference for TBA G4 DNA over the non-G4 control DNA is less than that observed for any of the parallel-stranded G4 substrates. In the presence of LiCl, the binding affinity did not change substantially for either substrate (TBA = 139 ± 17 nM, non-G4 control = 310 ± 40 nM). In this regard, hRev1^330–833^ did not bind to the anti-parallel G4 structure formed by the TBA motif in a manner that was distinct from the non-G4 control.

Similar binding studies for hRev1^330–833^ were also performed using the primer-template ds-DNA G4 and non-G4 substrates, and the observed values for equilibrium dissociation constants are summarized in Supplementary Table S3, and the corresponding binding curves are shown in Supplementary Figure S9. In general, the primer-template substrates were observed to follow the same trend as that seen with the ss-DNA substrates. In summary, the binding trends observed for hRev1 and the parallel-stranded G4 substrates did not seem to carry over to other G4 folds, at least *in vitro*.

### Chemical footprinting mapped the G4 DNA interacting residues in the G-loop and the insert 2 region of the human Rev1 protein

**Figure 2.**
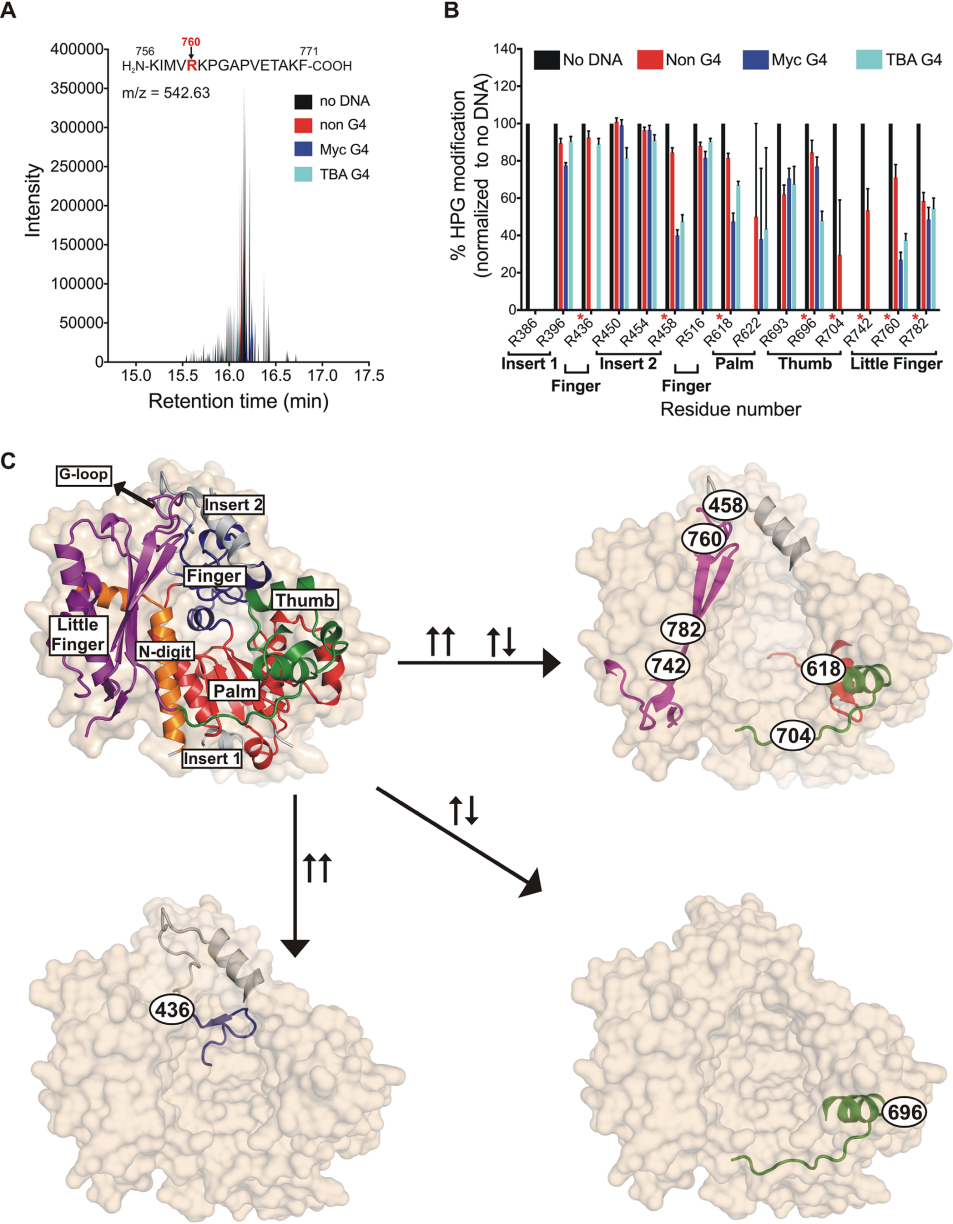
G4 DNA protects certain arginine residues on hRev1 from reactivity with HPG. (**A**) A representative total ion chromatogram is shown for a peptide (sequence shown above the plot) containing HPG-modified Arg760 following incubation with HPG alone (*black*), HPG + non-G4 DNA (*red*), HPG + Myc-G4 (*blue*) or HPG + TBA G4 (*cyan*). (**B**) For each of the 15 HPG-modified arginines detected, the fraction of modified arginine to total arginine was calculated and values across all samples were normalized to the ‘no DNA’ sample. The location of each arginine in the hRev1 protein catalytic domain (palm, finger, etc.), as well as the residue number, is indicated below the X-axis. The arginines highlighted in panel C are marked in *red* asterisks. Values shown represent the mean ± range (*n* = 2). (**C**) The structure of hRev1^330–833^ (PDB 3GQC) is shown as a molecular surface (*tan*; semi-transparent, in the background), as well as a cartoon showing the secondary structural elements. The domains are labeled and colored as: N-digit (*orange*), finger (*dark blue*), thumb (*green*), palm (*red*), little finger (*magenta*). The insert-1 and insert-2 regions are colored *light grey*. Position of the G-loop in the little finger domain is marked. The HPG-modified arginine-containing peptides protected specifically after incubation with Myc DNA (↑↑), TBA DNA (↑↓), or common to both (↑↑ ↑↓) are shown (↑↑ = parallel G4, ↑↓ = anti-parallel G4). Position and identity of each protected arginine is shown as the residue number inside a black oval.

We next sought to investigate the molecular features responsible for hRev1 interactions with G4 DNA by using chemical footprinting. We subjected hRev1 to chemical modification by *p*-hydroxyphenyl glycol (HPG) either in the presence of G4 or non-G4 DNA substrates. Similar treatment was also performed on protein samples without DNA to serve as a control. After the HPG reactions were quenched, the samples were subjected to tryptic digestion followed by LC–MS analysis of the resulting peptides (Supplementary Table S4 and Figure 2A). After identifying peptides containing HPG-modified arginines across all samples, the extent of HPG-modification at every arginine was calculated and expressed as a fraction of total occurrence of peptides that contain that arginine residue (Figure 2B). A decrease in the relative abundance of the HPG-modified peptide signaled protection from chemical reactivity attributed to the presence of the DNA substrate. By comparing results for non-G4, Myc G4 and the TBA G4 DNA, we hoped to detect regions of the enzyme that specifically interact with the structured G4 substrates.

We identified 15 arginine-containing peptides from the core polymerase domains of hRev1 that were modified by HPG. Residues R436, R458, R618, R742 and R760 were all more protected from HPG reactivity in the presence of G4 DNA than the non-G4 counterpart (Figure 2B). R436 was completely protected from modification exclusively in the Myc-G4 DNA bound sample but was HPG-modified in all other samples. Based on the crystal structure of hRev1 in ternary complex with primer-template DNA (11), R436 resides on the C-terminal end of the αD helix in a loop that sits on the backside of the finger domain (Figure 2C). Similar to R436, R458 was protected only in the G4 DNA samples (50–60% in both Myc and TBA) but not in the non-G4 DNA samples (∼15%). The R458 residue is located on the N-terminal side of the αE helix in the insert-2 motif (Figure 2C). Insert-2 is comprised of 54 amino acids that were shown to serve as a ‘flap’ over a hydrophobic pocket that is formed by residues from the N-digit and finger domains, as well as a short motif in the little finger domain called the ‘G-loop’ (11). Importantly, this pocket houses the ejected template base, helping endow Rev1 with its unusual protein-template mechanism of nucleotide selection. Two other residues selectively protected from HPG by G4 DNA were R742 and R760, each of which reside in the little finger (or palm-associated domain). R760 helps to form the G-loop, whereas R742 is part of the linker region connecting the thumb and little finger domains.

Another residue that was less reactive with HPG in the presence of G4 substrates was R618, which resides in the palm domain and interacts with the primer backbone (11). The palm domain residue R618 was protected in both G4 DNA bound samples but was more protected in the Myc (∼55%) compared to TBA (∼33%). We note that the chemical footprinting reaction reported here was performed with ssDNA for all substrates. In this respect, the diminished reactivity of R618 may be related to how the quadruplex structure is accommodated by hRev1 when the enzyme is not localized to the primer-terminus. Finally, the R696 residue in the thumb domain was more protected by TBA G4 DNA than the Myc G4 DNA structure (Figure 2C, compare ∼50% protection by TBA with ∼23% protection by the Myc substrate), which could demarcate either a different binding mode or altered conformational dynamics of the two G4 substrates near this site.

### Mutating residues that form the hydrophobic pocket selectively reduced the affinity of hRev1 for G4 DNA

**Figure 3.**
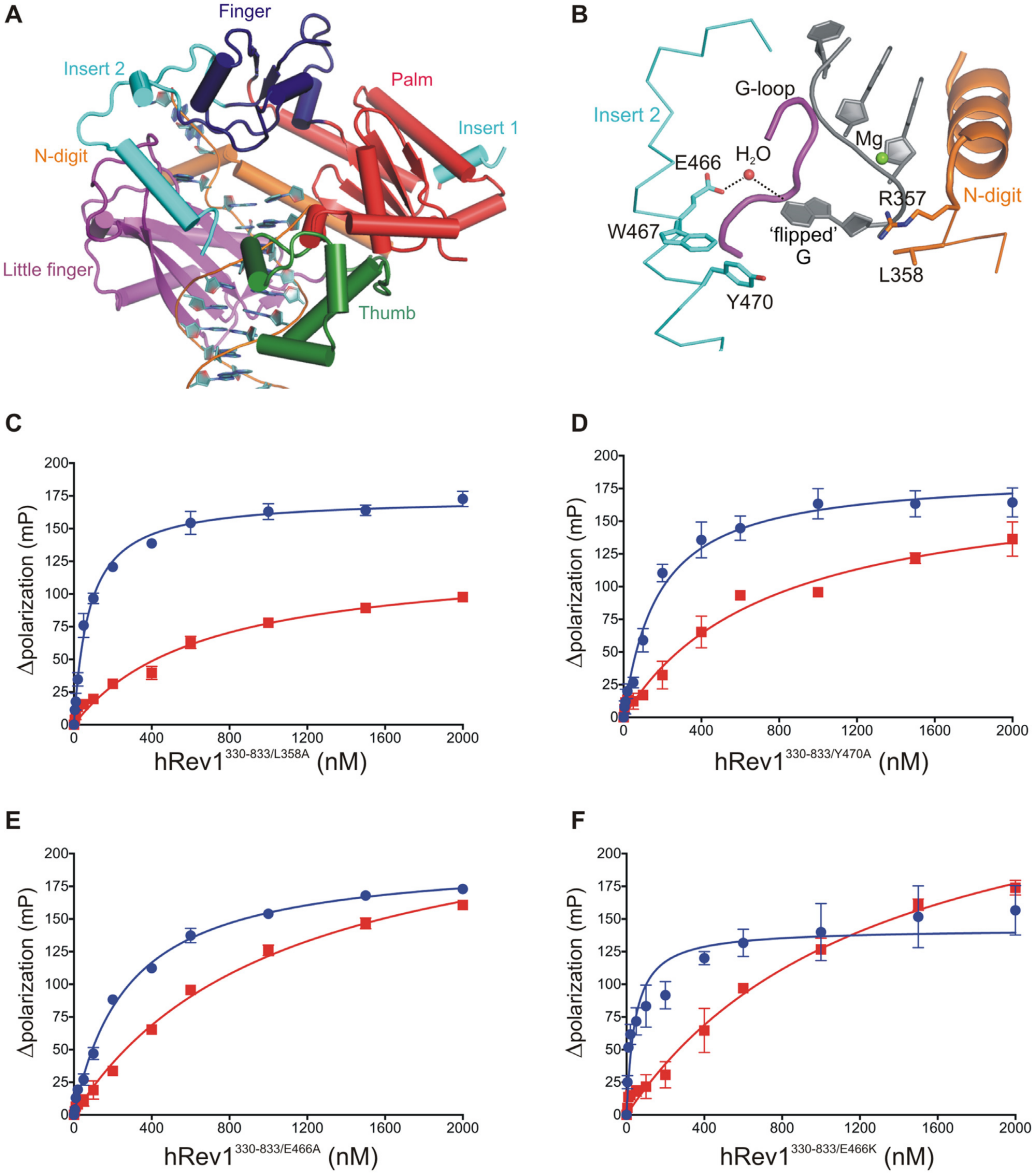
Mutations in insert-2 alter hRev1 binding to G4 DNA. (**A**) The hRev1^330–833^ ternary complex is shown (PDB 3GQC). The finger (*blue*), palm (*red*), thumb (*green*), and little finger (*magenta*) domains found in all Y-family polymerases are noted. (**B**) A hydrophobic pocket comprised of residues from insert-2 and the little finger domain is the site for positioning the ‘flipped’ guanine of the template during catalysis. L358 from the N-digit facilitates the eviction of the template guanine, which is stabilized through a water-mediated bridge with residue E466 in insert-2. Additionally, residues W467 and Y470 (also from insert-2) provide the hydrophobic stacking interactions for the evicted base. Binding affinity for G4 (*blue*) and non-G4 (*red*) DNA was measured for the (**C**) L358A, (**D**) Y470A, (**E**) E466A and (**F**) E466K hRev1 mutant proteins. The measured binding constants are listed in Table 3. Reported values represent the mean ± SD (n = 3).

We were intrigued by the clustering of residues near the hydrophobic pocket that were protected from HPG reactivity in the presence of G4 substrates. This part of Rev1 is structurally distinct from other TLS pols and is directly involved in keeping the nascent template base extrahelical (Figure 3A). We also recognized that insert-2 is unique to multi-cellular animals (Supplementary Figure S10), which have displayed greater dependence on Rev1 function than yeast during G4 replication. We hypothesized that mutating amino acids from insert-2, as well those in and around the template binding pocket, would selectively disrupt hRev1 interaction with G4 DNA substrates. To test this idea, we performed site-directed mutagenesis on hRev1 – concentrating our efforts on mutating residues that are in close proximity to the ejected template base (Figure 3B).

The following residues of hRev1^330–833^ were targeted to generate mutant proteins: L358A, Y470A, E466A and E466K. These mutant proteins, along with the wild-type hRev1^330–833^, were purified to homogeneity (Supplementary Figure S2). The mutations did not seem to cause any deleterious effects on the structural integrity of the protein, as witnessed by almost identical circular dichroism spectra of the purified proteins (Supplementary Figure S2). To examine the effect of mutating residues in the hydrophobic pocket of hRev1 on G4 selectivity, we measured the *K*_D,DNA_ values for mutant enzyme binding the Myc 14/23 G4 DNA substrate and a non-G4 DNA control. We began by mutating the leucine residue responsible for ejecting the template base into the hydrophobic pocket (L358). The L358A mutant could still bind to both G4 and non-G4 substrates (Figure 3C). The *K*_D,DNA_ value for the G4 substrate increased 2-fold, from ∼40 nM for wild-type hRev1 to ∼80 nM for the L358A mutant enzyme. The *K*_D,DNA_ value for the non-G4 substrate decreased very slightly, from ∼900 nM for wild-type hRev1 to ∼700 nM for the L358A mutant enzyme. The overall effect of the L358A mutation was to slightly diminish the relative preference for G4 DNA relative to wild-type enzyme (Table 3).

**Table 3.** Equilibrium dissociation constants for mutant hRev1^330–833^ enzyme binding to ss-Myc-14/23 and non-G4 DNA substrates^a^

	*K*_D,DNA_		Fold preference for G4 DNA
	Non-G4 (nM)	G4 (nM)	(*K*_D,non-G4 DNA_/*K*_D,G4 DNA_)
L358A	660 ± 140	77 ± 8	9
E466A	1100 ± 130	280 ± 20	4
E466K	1300 ± 300	43 ± 16	30
Y470A	740 ± 180	180 ± 30	5

^a^The single-stranded Myc 14/23 G4 and non-G4 DNA substrates were used to measure the binding affinities reported here. Data represent the mean ± SD (*n* = 3).

Next, we examined the role of insert-2 in governing hRev1 interactions with G4 DNA. We wanted to evaluate the role of aromatic amino acid side-chains located in insert-2 since these types of residues could interact with the planar face of G-quartets and influence binding selectivity. Previous structural reports have shown that aromatic side-chains from residues forming the hydrophobic pocket interact with extrahelical template guanine and DNA adducts (11,33). We selected two residues in the insert-2 helix, W467 and Y470, that could be important for similar stabilizing interactions with G4 DNA. Multiple attempts to purify the W467A mutant were not successful, as very little soluble protein was recovered. The Y470A mutant enzyme, on the other hand, expressed well and was readily purified. We discovered that mutating Y470 disrupted binding to the Myc 14/23 G4 substrate but did little to alter the affinity of the mutant protein for the non-structured control DNA (Figure 3D and Table 3). The *K*_D,DNA_ value for the G4 substrate increased around 4-fold, from ∼40 nM for wild-type hRev1 to ∼180 nM for the Y470A mutant enzyme, indicative of less affinity for the structured substrate. The *K*_D,DNA_ value for the non-G4 substrate decreased very slightly, from ∼900 nM for wild-type hRev1 to ∼740 nM for the Y470A mutant enzyme. Compared to the L358A mutant, mutating Y470 to alanine had a more pronounced effect on the relative preference of hRev1 for G4 DNA, diminishing G4 selectivity by ∼5-fold.

A stronger effect on selective binding to G4 was observed when we mutated E466 to alanine (E466A). The preferential binding to G4 substrates decreased ∼6-fold, primarily due to an almost 8-fold decrease in affinity for the G4 substrate (Figure 3E and Table 3). The *K*_D,DNA_ value for the G4 substrate increased to almost 300 nM for the E466A mutant, while the *K*_D,DNA_ value for the non-G4 substrate was similar to that observed for wild-type hRev1. The side-chain of E466 is connected to the template guanine via a hydrogen bonding lattice that involves a solvent water molecule. We reasoned that mutating E466 to lysine (E466K) would restore the hydrogen bonding pattern necessary for interactions with the G4 substrate. In support of this notion, the E466K mutant exhibited binding affinities that were almost identical to wild-type hRev1 (Figure 3F and Table 3). The striking results with the E466 mutants combined with the results for the Y470A mutant confirmed the importance of insert-2 for selective binding to G4 substrates by hRev1.

### Insert-2 mutations that alter G4 binding properties did not influence hRev1 polymerase activity in a G4-specific manner

**Figure 4.**
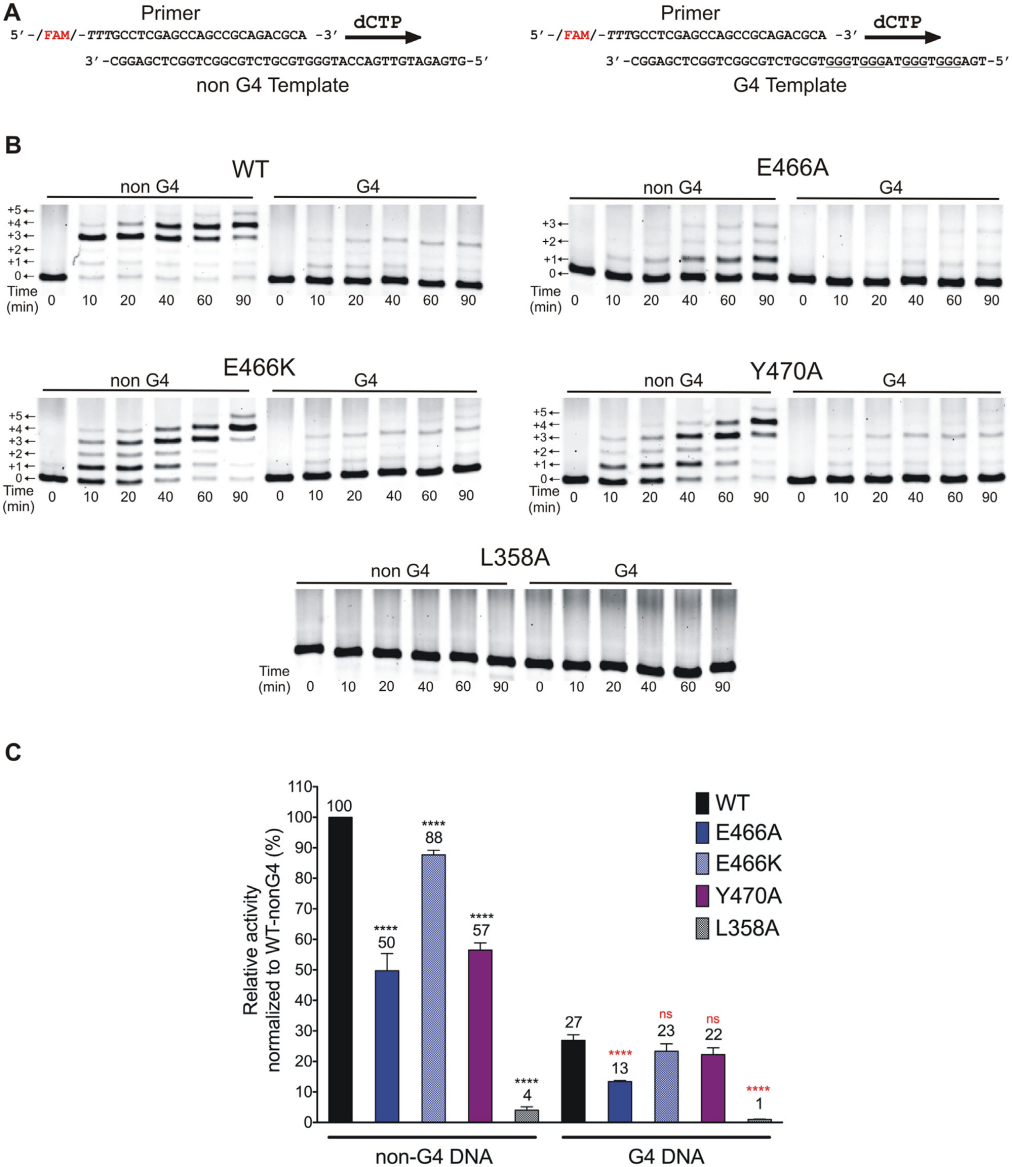
Alterations in the catalytic activity of hRev1 mutants were largely unrelated to whether G4 was present in the template strand. (**A**) Schematic illustration of the primer-template DNA substrates. Arrows indicate the direction of primer extension by multiple insertions of dCMP across the template during the time course. (**B**) Representative gel images for the time course monitoring dCMP insertion by the wild-type and mutant hRev1^330–833^ proteins (50 nM) on non-G4 or G4 DNA substrates (200 nM) in a buffer containing 100 mM KCl. The positions of the primer and additions of dCMP (+1, +2, etc.) are marked beside the gel images. (**C**) Relative enzyme activity for each protein is shown for the non-G4 and G4 DNA substrates. Relative activity is reported as a percentage of the activity of wild-type hRev1^330–833^ on the non-G4 DNA substrate. The mean value for relative activity for each protein is shown at the top of its corresponding bar. Reported values represent the mean ± SD (n = 3). Statistical significance was assessed by performing a one-way ANOVA with multiple comparisons, and the *P*-values were calculated for each comparison. **** indicates *P* < 0.0001 for comparisons between mean values of each mutant enzyme with that of the wild-type on non-G4 substrate, **** indicates *P* < 0.0001 for comparisons between mean values of each mutant enzyme with that of the wild-type on the G4 substrate. ns; non-significant (*P*> 0.05).

Next, we proceeded to test the cytidyl transferase activity of hRev1^330–833^ wild-type and mutant proteins on both non G4 and G4 DNA. We prepared two 23/42-mer primer-template DNA substrates – one with a template containing the Myc 14/23 sequence and one with a non-G4 control sequence (Figure 4A). As reported previously (17), wild-type hRev1^330–833^ is able to extend the nascent primer strand by incorporating multiple dCMPs on both non-G4 and G4 DNA substrates (Figure 4B). Wild-type hRev1 readily extended the primer across the run of three guanines on the non-G4 template, even adding a fourth and fifth nucleotide to the primer at later time points (Figure 4B). Appreciable activity on the G4 DNA substrate was observed with wild-type hRev1. However, the efficiency of the reaction was reduced (∼3-fold) as compared to non-G4 DNA (Figure 4B, quantified in 4C). The enzyme added dCMP opposite the first run of three guanines of the quadruplex, but then seemed to stall. Moreover, there is a large fraction of unreacted substrate left over even after 90 min, a trend that was not observed with the non-G4 substrate.

Among the mutant enzymes, the L358A mutant protein was found to have <5% of the wild-type activity (Figure 4B and C). The E466A mutant enzyme was the second most affected in terms of catalytic activity. As seen in Figure 4B (second panel), although it did show measurable activity on the non-G4 substrate, it was only ∼50% that of wild-type, and the amount of unreacted primer left over at the end of 90 min was much greater than wild-type for the non-G4 substrate—indicative of less substrate turnover for the E466A mutant enzyme. The activity of the E466A mutant was similarly reduced by ∼50% on the G4 substrate compared to wild-type on the same substrate. By way of comparison, the mutant E466K was almost as active as wild-type hRev1 on both substrates (∼10% reduction). The G-loop mutant Y470A, on the other hand, showed significantly reduced activity on the non-G4 substrate, when compared to wild-type, while the activity on G4 DNA was comparable to that of wild-type enzyme. Overall, the results of the polymerase activity assays did not provide evidence to suggest that the mutant enzymes lost catalytic activity specifically on G4 DNA substrates.

### G4 replication is mutagenic and sensitive to quadruplex stabilization in hRev1-deficient cells

By measuring changes in the specificity constant (*k*_cat_/*K*_M,dNTP_) as a function of how close the primer terminus was positioned relative to a G4 motif in the template strand, we were previously able to show that the Y-family member hpol η is more accurate and efficient than the replicative enzyme hpol ϵ when it comes to nucleotide incorporation on a G4-containing substrate (34). hRev1, on the other hand, exhibited substantial decreases in catalytic efficiency when we compared the specificity constants for G4-containing substrates with the primer terminus positioned adjacent to the first tetrad-associated guanine in the Myc 14/23 template sequence (17). While the role for hRev1 catalysis may be minimal or dispensable for suppressing mutations near G4 motifs, the strong preference of hRev1 for binding to parallel-stranded G4 DNA substrates in biochemical studies combined with the known impact of the Rev1 C-terminus on G4 replication efficiency led us to explore the effects of hRev1 deletion on the accuracy of G4 replication in human cells. We employed the *supF* forward mutagenesis assay, which has been used to evaluate the replication past many types of DNA adducts and other unusual template structures (35). To investigate the role of hRev1 in replication of G4 motifs, we inserted the Myc-derived G4-forming sequence upstream and within the *supF* gene of the pSP189 plasmid (Supplementary Figure S4). The *supF* gene, which codes for the amber-suppressor tRNA in the *lacZ* gene, served as a readout for any mutations in and around the G4-insert with the unmodified pSP189 plasmid serving as the control.

**Figure 5.**
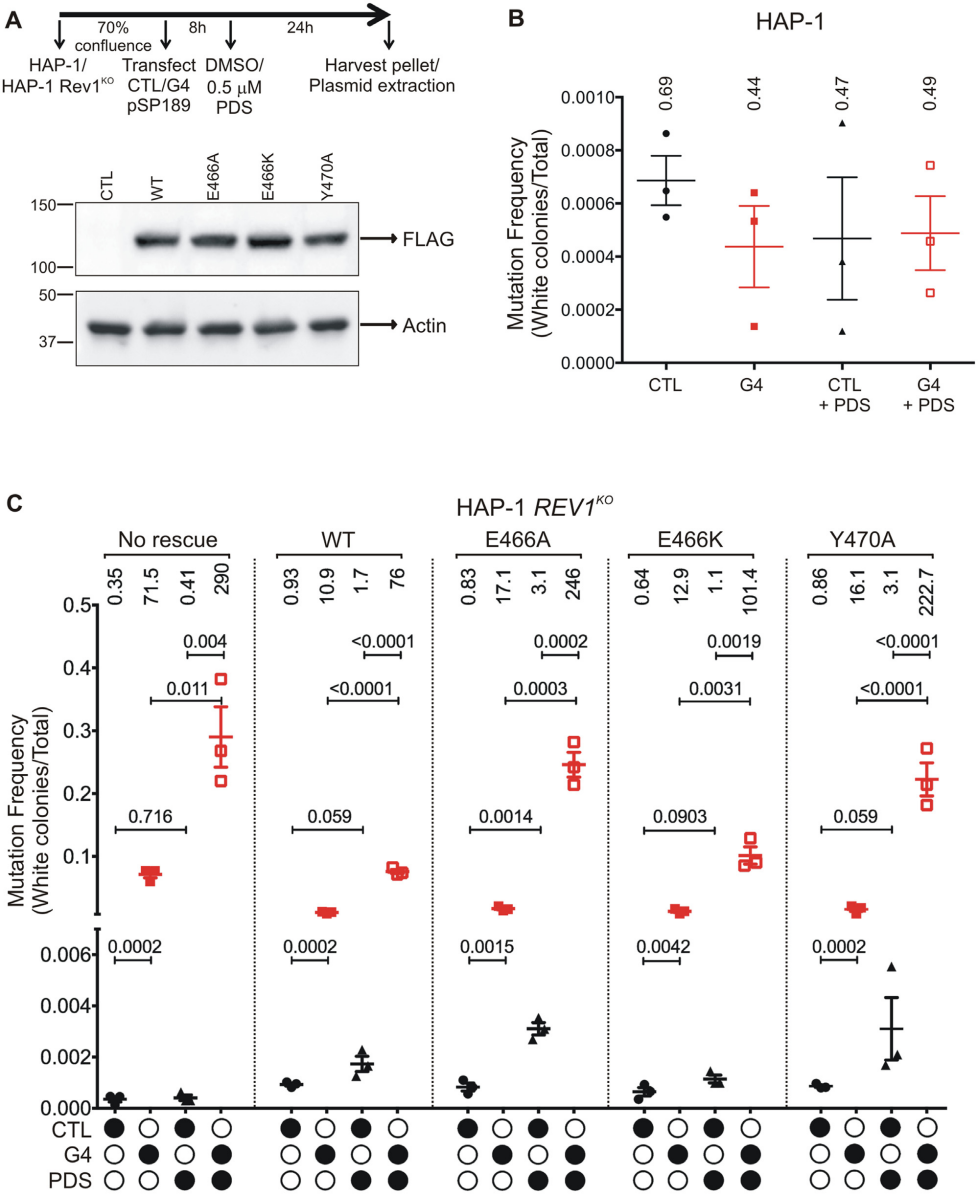
hRev1 plays an important role in suppression of mutations at sites containing G4 DNA sequences. (**A**) An overview of the *supF* mutagenesis experiments is shown. Briefly, wild-type HAP-1 and *REV1*^KO^ cells were transfected with either the unmodified pSP189 plasmid (control) or the pSP189 plasmid containing a Myc-G4 sequence. This was followed by treatment with either vehicle (DMSO) or 0.5 μM pyridostatin (PDS). The cells were cultured for an additional 24 h. Then plasmids were extracted and used for blue-white screening by transformation into *E. coli* MBM7070 cells, followed by plating on to LB-Agar plates containing X-Gal and IPTG. Complementation *supF* mutagenesis experiments were performed similarly, using *REV1*^KO^ cells co-transfected with SFB-tagged wild-type or mutant hRev1^1–1251^. A Western blot of whole-cell lysates of *REV1*^KO^ cells showing the relative protein expression of each of the transiently transfected SFB-tagged hRev1^1–1251^ protein is shown. *CTL* - control (untransfected); *WT* - cells transfected with wild-type hRev1^1–1251^; *E466A* - cells transfected with the E466A mutant hRev1^1–1251^; *E466K* - cells transfected with the E466K mutant hRev1^1–1251^; *Y470A* - cells transfected with the Y470A mutant hRev1^1–1251^. The corresponding uncropped immunoblot images are shown in supplementary Supplementary Figure S5. The number of blue and white colonies were counted and the mutation frequency was calculated for each condition. A minimum of 10,000 colonies were counted for each replicate. Experiments were performed in triplicate and the results were plotted as mean ± SD (*n* = 3). The mutation frequencies calculated for the (**B**) HAP-1 and (**C**) *REV1*^KO^ cells are shown. *CTL* - cells transfected with control pSP189 plasmid; *G4* - cells transfected with the G4-pSP189 plasmid; *PDS*—cells treated with 0.5 μM PDS. For the complementation experiments in the *REV1*^KO^ cells, the identity of the hRev1 variant used is noted above the data. The mean value of mutation frequency (× 10^−3^) is shown above each set of data points. Statistical significance was assessed by performing a pair-wise Student's *t*-test, and the *P*-value calculated is shown above each pair.

Both wild-type and *REV1*^KO^ HAP-1 cells were grown to ∼70% confluence, followed by transfection with the control or G4-containing pSP189 plasmid either in the presence or absence of 0.5 μM PDS or DMSO (vehicle control). The cells were cultured for 24 h post-transfection, harvested, and the replicated plasmids were retrieved (Figure 5A). The retrieved plasmids were then used to transform electro-competent *E. coli* MBM7070 cells, followed by plating the transformants on LB-Agar plates containing X-Gal and IPTG. After colonies appeared, the plates were incubated at 4°C for 15–24 h to allow the blue color to intensify. Finally, the blue and white colonies on each plate were counted using the Fiji version of the ImageJ software (Figure 5A). Experiments were performed in triplicate, and the mutation frequency was calculated by plotting ratio of the number of white colonies to total number of colonies counted for each experimental condition.

The control and G4 plasmids were replicated with the same accuracy in wild-type HAP-1 cells (Figure 5B). The corresponding values for background mutation frequency were calculated to be 4.4 × 10^−5^ and 9.6 × 10^−5^ for the control and G4 plasmids, respectively. The addition of PDS did not change the mutation frequency for either plasmid in wild-type cells (Figure 5B). Replication of the control plasmid in *REV1*^KO^ cells was comparable to wild-type HAP-1 cells and was unaffected by the addition of PDS (Figure 5C). There was a dramatic 200-fold increase in mutation frequency when the G4-containing plasmid was replicated in *REV1*^KO^ cells (Figure 5C). This effect was further amplified by the addition of PDS, which elicited a further 4-fold increase in mutation frequency relative to what was observed for the G4 plasmid without PDS. The mutation frequency for the PDS-stabilized G4 plasmid was >700-fold higher than that observed for the control plasmid in the presence of PDS (Figure 5C). These results clearly demonstrated the central role of hRev1 in preventing mutations at G4 DNA sites.

Complementation experiments were then performed to determine if re-expressing hRev1 would rescue the mutagenic replication phenotype observed in *REV1*^KO^ cells. Equal expression of wild-type and mutant hRev1 proteins was confirmed by immunoblotting (Figure 5A). Transient re-expression of wild-type hRev1 decreased the mutation frequency for the G4-containing plasmid ∼7-fold (Figure 5C). Replication of the G4 plasmid in the presence of PDS decreased 4-fold when wild-type hRev1 was re-introduced to the *REV1*^KO^ cells (Figure 5C). The E466A mutant protein reduced the G4 mutation frequency ∼4-fold, which is not that different from the wild-type enzyme (Figure 5C). However, there was a marked inability of the E466A mutant to rescue the PDS-induced increase in mutation frequency observed for *REV1*^KO^ cells (Figure 5C). PDS-induced mutations in the G4 plasmid were diminished when the E466K hRev1 mutant was expressed – reducing the mutation frequency almost 3-fold from that observed for *REV1*^KO^ cells (Figure 5C). In this regard, restoring the hydrogen-bonding capacity at position 466 facilitated more accurate bypass of a PDS-stabilized G4 structure. Expression of the Y470A mutant resulted in trends that were very similar to the E466A mutant. The mutation frequency for the G4 plasmid was reduced ∼4-fold in cells expressing the Y470A mutant compared to *REV1*^KO^ cells, but hRev1 Y470A was not able to suppress the increase in mutation frequency observed for the G4 plasmid when cells were treated with PDS. In summary, complementation with wild-type and the mutant hRev1 enzymes partially suppressed the mutagenic G4 replication observed in *REV1*^KO^ cells. Re-expressing either wild-type or E466K hRev1 was able to largely eliminate the increase in mutation frequency observed for cells treated with PDS. G4-defective hRev1 mutants (E466A and Y470A) were unable to suppress PDS-induced mutations on a G4-containing plasmid. The importance of residues involved in selective G4 binding was most evident when quadruplexes were stabilized by PDS.

### Loss of hRev1 activity resulted in an increased number of deletions occurring upstream of G4 DNA

**Figure 6.**
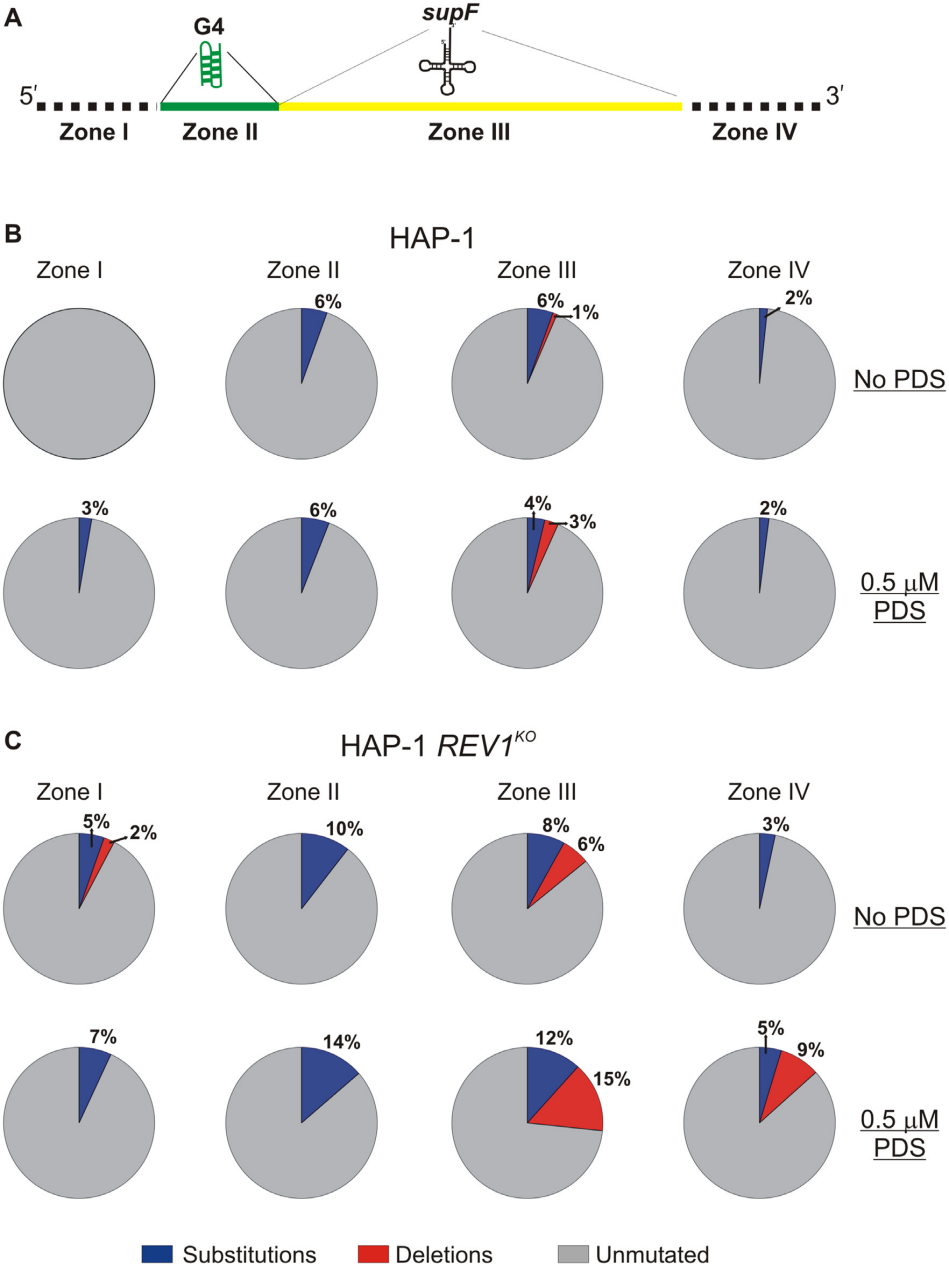
The presence of functional Rev1 determines the types of mutations near a quadruplex sequence. (**A**) A schematic representation of the DNA sequence surrounding the *supF* gene in the pSP189-G4 plasmid is shown. The region analyzed for mutations was divided into four ‘zones’: Zone I (dotted line) includes 26 bp to the 5′-side of the G4 sequence, Zone II (*green*) includes the G4 sequence derived from the c-Myc promoter, Zone III (*yellow*) includes the 85 bp *supF* tRNA coding sequence, and Zone IV (dotted line) 30 bp sequence on the 3′-side of the *supF* gene. The sequences of the mutated plasmids harvested from white colonies are reported in supplementary information (Supplementary Figures S6 and S7). The distribution of mutations (base substitutions and deletions) on the G4 plasmid obtained for (**B**) HAP-1 cells and (**C**) *REV1^KO^* cells (±0.5 μM PDS) is reported for each zone. Ten plasmids extracted from white colonies from three replicates from each experimental condition were sequenced. A multiple sequence alignment of all the DNA sequences, including the original G4-pSP189 plasmid was performed using T-Coffee (21). At each position, the type of mutation - substitutions (*blue*) or deletions (*red*) were marked and summed over all 10 sequences per condition. Non-mutated positions (*grey*) were also counted similarly. Each pie-chart represents the percentage distribution of substitutions, deletions or non-mutated bases for each of the four zones

We extracted plasmids from mutant (white) colonies and sequenced the products in order to determine the nature and position of mutations. This was done for the white colonies from both the HAP-1 cells (Supplementary Figure S6) as well as the *REV1^KO^* cells (Supplementary Figure S7). To help us understand hRev1-dependent changes in the mutation profiles, we divided the plasmid sequence surrounding the *supF* gene into four ‘zones’ (Figure 6A). We defined zone I as the sequence occurring on the 5′-side of the G4 motif. Zone II included the 18-mer Myc G4 sequence (shown in *green* on Figure 6A), while zone III was defined as the entire *supF* coding region (shown in *yellow* on Figure 6A). Zone IV was defined as the sequence on the 3′-side *supF* coding region. In this way, we could evaluate the effect of hRev1 activity as the replisome approached the quadruplex, as well as mutations within the actual G4 motif or those occurring after the replisome had traversed the sequence. Results from DNA sequencing of 10 white colonies from each experimental condition were compiled, and the numbers and types of each mutation was expressed as a fraction of total number of bases in the four zones together. Plasmids were harvested from different biological replicates to help ensure reproducibility of the sequencing results.

In HAP-1 cells, the largest number of mutations were observed in zones II and III containing the G4 motif and the *supF* gene, respectively (Figure 6B). The addition of PDS did not greatly alter the identity of the mutations in zones II or IV for the parental cell line, but there was a shift towards more deletions in the *supF* gene (zone III). There were also base substitutions in zone I that were dependent on PDS treatment. Compared to wild-type HAP-1 cells, *REV1*^KO^ cells had a higher percentage of mutated bases in all zones with the largest fraction of mutations in *REV1*^KO^ cells being observed in the G4 (zone II) and *supF* (zone III) containing zones (Figure 6C). Both base substitutions and deletions were observed in zones I and III, whereas zones II and IV only had base substitutions. The addition of PDS increased the number of mutations in every zone, including a pronounced increase in deletions for zones III and IV (Figure 6C). When compared to HAP-1 cells, the *REV1*^KO^ cells exhibited a higher percentage of mutations, extending over a larger area of the DNA sequence, and PDS treatment strongly amplified this effect. In this regard, hRev1-deficiency resulted in less accurate copying of sequences surrounding the G4 motif and stabilization of the G4 structure exacerbated these defects even further.

## DISCUSSION

G-quadruplexes act as natural barriers to DNA replication and require the action of helicases and polymerases that have evolved to copy past these dynamic DNA structures (36). Rev1 has been identified as a key protein in this regard (9,10,16). The C-terminal domain of Rev1, which interacts with other TLS polymerases, was shown to be important for uninterrupted fork progression and corresponding maintenance of histone marks near G4 sites, with the catalytic activity of Rev1 being partially dispensable for these effects (9). The FANCJ helicase may actively recruit Rev1 to form a G4 repair complex via direct interaction through its PCNA-interacting protein (PIP) like domain (15). Likewise, Rev1 helps recruit other TLS pols that may assist in copying G4 motifs. From biochemical studies, we concluded that hRev1 could bind preferentially to G4 DNA with high affinity and could unfold it *in vitro* without the requirement of its catalytic activity (17), but the molecular basis for this activity has not been fully elucidated. Given the central role of Rev1 in replication at G4 DNA sites, we set out to identify the molecular determinants of the hRev1-G4 interface.

Results from our DNA binding studies demonstrated that hRev1 exhibits a strong preference for binding to parallel type of G4 structures like those formed by the Myc, Bcl2 and KRAS sequences, over the anti-parallel (TBA) or hybrid (hTelo4) G4 structures. Yet, even the anti-parallel and hybrid G4 structures were bound with modest preference over the non-G4 forming sequence. All three G4 folds share the stacked arrangement of planar tetrads but differ in the conformation of the backbone phosphates and loops/linkers that shape the non-planar grooves. Parallel-stranded quadruplexes have a double-chain reversal conformation for the loops between tetrad guanines. Anti-parallel G4 structures have loops that are either diagonal (across the tetrad) or edgewise (running along one side of the tetrad), which presents a different topology to G4 binding proteins. Another difference between G4 folds is the presence of *syn*-oriented guanines in anti-parallel-stranded structures, where as parallel-stranded structures seem to only have guanines with *anti* glycosidic bond angles. The exact reason hRev1 binds parallel-stranded G4s better than either an anti-parallel or hybrid fold is unknown.

The G4 helicase DHX36 selectively binds and unwinds parallel-stranded G4s (37,38). The selective binding of DHX36 to the parallel-stranded Myc G4 was attributed to the conformation of the loops connecting each run of guanines. DHX36 binding to the Myc G4 relies on a non-polar surface formed by the so-called DHX36-specific motif (DSM) that sits on top of the planar tetrad. The double-chain reversals of the Myc G4 structure allow residues I65, W68, Y69, and A70 in the DSM to approach the planar tetrad without interference (39). Positively charged residues in the oligonucleotide and oligosaccharide-binding (OB) fold of DHX36 hydrogen bond with the phosphate backbone and G4 residues on the Myc substrate. Other proteins possess RGG repeats that confer G4 specific binding properties (40). There is a single RGG sequence near the hRev1 N-terminus (residues 3–5). This region is not critical for the G4-selective binding properties of hRev1, as demonstrated by the fact that the polymerase core (a.a. 330–833) retains preferential affinity for G4 DNA substrates. Given the role for Y470 as a determinant of G4 binding, it seems likely that the double-chain reversal loop conformation adopted by parallel-stranded G4 structures is most conducive to the hRev1-G4 interaction.

The selective binding of hRev1 to G4 motifs that adopt a parallel-stranded fold is interesting. The replisome must traverse a genomic landscape that likely presents a menagerie of non-canonical DNA structures. Defects in hRev1 function could jeopardize maintenance of G4 motifs that readily adopt the parallel-stranded fold, such as those that exert regulatory control over *c-MYC* and *KRAS* oncogenes. The identification of a G4 motif in the *REV1* promoter capable of folding into a very stable, parallel-stranded quadruplex structure raises the intriguing possibility that a self-regulatory mechanism may be in place to control *REV1* gene expression. In such a scenario, hRev1-mediated maintenance of epigenetic stability surrounding G4 sites could, in turn, determine *REV1* expression. A similar G4-dependent regulatory loop was reported for the HELB helicase (41), as well as for poly (ADP-ribose) polymerase 1 (PARP1), a DNA repair protein of great clinical relevance that exhibits important G4 properties (42–44). Further study along these lines may uncover additional points of connectivity between proteins that act on G4 substrates and quadruplex-mediated regulation of gene expression.

Chemical footprinting identified residues near the hydrophobic pocket (where the nascent template base is secured) as being specifically protected by G4 DNA substrates. Mutations in insert-2 of hRev1 produced changes in G4 DNA binding properties that were subsequently shown to influence the accuracy of G4 replication in cells. The E466 residue in hRev1 forms a water-mediated contact with the evicted template dG base, stabilizing the base in the hydrophobic pocket. Mutating E466 to an alanine reduced the G4-binding preference of hRev1 dramatically compared to wild-type. The wild-type enzyme exhibited a >20-fold preference for G4 whereas the E466A mutant bound G4 DNA ∼4-fold more tightly than the non-G4 control. Subsequent analysis of *supF* mutagenesis results revealed that, in contrast to wild-type hRev1, expressing the E466A hRev1 mutant did not restore the accuracy of replication across PDS-stabilized G4 DNA. These findings are interesting because the E466A mutant possesses all of the domains and features needed for protein-protein interactions previously implicated in G4 maintenance, including the C-terminal domain known to recruit other TLS pols to sites of replication stress. Similar trends were observed with the Y470A mutant. In each case, the diminished selectivity for G4 DNA at the binding step that we observed in binding assays correlated with a marked decrease in the ability of replication forks to copy a PDS-stabilized G4 motif. Interestingly, the E466A and Y470A mutants behaved largely as wild-type enzyme in promoting a reduction in mutation frequency on the G4 plasmid in the absence of PDS, suggesting that G4 selective binding may not be critical for TLS across G4 motifs unless they are stabilized in some way. A previous study did not find a correlation between G4 stability and Rev1 activity in avian cells (10). Additional studies with hRev1 could help clarify the relationship between Rev1 action and G4 structures stabilized either by compounds, such as PDS, or by proteins that specifically bind G4 structures.

Validation of the idea that G4 selective binding by hRev1 can influence the outcome of replication in cells was provided by experiments with the E466K mutant. This mutation completely restored wild-type G4 binding properties, as evidenced by the 30-fold preference of the Myc 14/23 G4 substrate over the non-G4 control (Table 3). The E466K mutant also exhibited polymerase activity closely comparable to wild-type hRev1 (Figure 4). Expression of the E466K hRev1 mutant reduced the mutation frequency for the G4 plasmid in both the presence and absence of PDS, similar to wild-type enzyme (Figure 5). With that said, the mutation frequency for the G4 plasmid in the complementation experiments was still higher than that of the parental hRev1-proficient HAP-1 cell line. So, while the transient expression of hRev1 in the *REV1*^KO^ line did not fully recapitulate the replication program of HAP-1 cells, re-expressing both wild-type and mutant hRev1 resulted in decreased mutation frequencies for the G4 plasmid in the absence of PDS, but only wild-type and the E466K mutant were able to diminish the number of mutations in the G4 plasmid when PDS was present. These experiments provide support for the idea that insert-2 is an important determinant of G4 selective binding by hRev1 and that this is a key step in facilitating accurate replication of stabilized G4 motifs.

A recent time-lapse crystallographic study of yeast Rev1 found that the ejection of the template base preceded nucleotide binding and that the templating base remained extrahelical even after nucleotide incorporation (12). Hydrogen bonding between the backbone amides of M685/G686 in the G-loop of yeast Rev1 help keep the template base extrahelical. For hRev1, the E466 side-chain combines with the backbone amide of G775 to stabilize the template base in the hydrophobic pocket. It is possible that hRev1 may also keep the template base extrahelical after dCMP insertion. The ability to maintain an extrahelical template base through interactions involving insert-2 could become especially important for maintaining an unfolded state during replication across G4 motifs. This may be why the E466A and Y470A mutants could not suppress mutagenic replication of the PDS-stabilized G4. Lacking key contacts with the template base, E466A and Y470A would not be able to keep tetrad-associated guanines in an extrahelical state when the G4 structure is stabilized by PDS.

Rev1 acts as a scaffold for recruitment of the other Y-family polymerases, and the Rev1-polζ mutasome is a major source of mutations for eukaryotes (45). The Rev1 protein-template mechanism of nucleotide selection allows for bypass of abasic sites and other lesions (46). Since G4 structures are reportedly most abundant during S-phase (5), there is a logical rationale for Rev1 and other TLS enzymes in facilitating bypass of difficult-to-copy sequences like G4 motifs. However, this is probably not all there is to the story of Rev1 and G-quadruplex maintenance. While the major focus of our study was unmodified G4, it is possible (if not likely) that Rev1 participates in replication and repair of modified G4 motifs. Guanine-rich sequences, specifically tandem guanines, are more susceptible to direct oxidation, as well as oxidation through electron hole migration (47). The formation of 8-oxo-7,8-dihydroguanine (8-oxoG or OG) and other products of guanine oxidation can, of course, increase the risk of mutation, but an emerging feature of guanine oxidation, specifically oxidation of tetrad-associated guanines, is related to the fact that there is an epigenetic-like relationship between OG in gene promoters and transcriptional regulation of DNA repair genes (47). More than half of all G4 motifs in gene promoters have a fifth run of guanines (the ‘spare tire’) (48). Oxidation of guanine and formation of an abasic site can both disrupt G4 structures by interfering with the Hoogsteen hydrogen-bonding pattern of tetrads. The extra run of guanines can sustain the G4 fold by replacing the damaged region, which is then presented as a substrate for DNA repair factors and other proteins (47) This ‘writing’ of OG into the G4 motif serves as a signal to up-regulate DNA repair genes. The *REV1*, *POLZ*, *POLH* and *POLK* genes were included amongst the list of genes with promoter G4 sequences, establishing the possibility that oxidative damage to G4 motifs up-regulates not only repair factors but TLS pols important for tolerance of DNA damage. As far as we know, there is no evidence that TLS pols are recruited to damaged G4 structures or just G4 structures in general. Given the results for *REV1*^KO^ HAP-1 cells reported here, as well as previous experiments with *rev1* mutant DT40 cells from the Sale laboratory, DNA damage does not seem to be required for Rev1-related effects to manifest near G4 motifs. However, given the importance of Rev1 in DNA damage tolerance mechanisms, it seems plausible that there is a role for Rev1 in facilitating TLS across G4 motifs containing abasic sites or oxidized bases.

The interplay between oxidized guanines in G4 motifs and transcriptional regulation of DNA repair is intriguing. Multiple routes to transcriptional activation have been linked to formation of OG in promoter G4s. Base excision repair (BER) involving the OGG1 glycosylase and the APE1 endonuclease was demonstrated to act on OG lesions near G4 motifs (49). Formation of the abasic intermediate ‘unmasks’ putative quadruplex sequences (PQS), allowing these motifs to adopt the four-stranded G4 structure and impacting transcriptional profiles (49). It is possible that Rev1 has a previously unrecognized role in error-prone BER at G4 sites. Rev1 is known to possess properties consistent with a role in BER (e.g., low processivity, the ability to use a gapped/abasic substrate, and weak 5′-dRP lyase activity (50). Moreover, mouse fibroblast cells lacking pol β rely on Rev1 for MMS-induced mutagenesis, leading to the suggestion that Rev1 could be a backup pol for BER (51). There is also a demonstrated role for Rev1 in promoting C to G transversions during somatic hypermutation of immunoglobin (Ig) genes in germinal center B cells of mice, presumably through insertion of CMP opposite abasic sites (52). The richness of G4 motifs in Ig loci may provide a mechanism for recruiting Rev1 to sites where deoxyuridine residues have been introduced by activation-induced cytidine deaminase. It will be of interest to determine if there is an enhanced role for Rev1 in BER at G4 motifs, as opposed to the classical TLS model for this enzyme. Given the limited processivity of Rev1, recruitment of other TLS pols through interactions with the Rev1 C-terminal domain is likely a critical part of successful G4 replication. Indeed, loss of the Rev1 C-terminal domain failed to rescue defects in G4 replication in DT40 cells (9). Yet, in spite of a clear role for TLS pols in G4 replication, there are aspects of this function that continue to be clarified. This was recently illustrated by the finding that Zuo1 blocks Rev1 binding to G4s that form near UV damage, favouring nucleotide excision repair of the UV-induced lesion (53). The Y-family members pols η and κ participate in prevention of G4-induced genomic instability (54). This function probably depends on interactions with the Rev1 C-terminal domain, but the exact partitioning of TLS activity or redundancy in facilitating synthesis past G4 sites (damaged or not) is an area that still requires some clarification.

Rev1 is strictly a eukaryotic polymerase, as bacterial and archaeal genomes are not known to encode an equivalent protein-template-directed polymerase. At least some of the G4 selective properties of hRev1 reside in sequence elements that are not conserved in Rev1 from yeast or plants (Supplementary Figure S10). Alignment of Rev1 protein sequences revealed conservation of insert-2 in insects, fish, reptiles, amphibians, birds, and mammals (Supplementary Figure S10). The functional relevance of Rev1 to G4 replication may be limited to vertebrates based on studies in yeast, but the reason for this is unknown. G-quadruplex forming sequences are enriched in regulatory regions of genomes from all kingdoms (55–59) and G4-induced genomic instability can occur in any organism lacking certain enzymes (e.g., Topo1 and Pif1 in yeast, *recF* proteins in *Radiococcus radiodurans*, and RecQ helicases in animals and plants). A recent comparison of G4 type and distribution in 12 model organisms revealed a multitude of interesting trends (59). For example, two-tetrad forming sequences were found to comprise a larger fraction of G4 motifs in *E. coli*, *Saccharomyces* and *Arabidopsis* genomes than either human or mouse genomes, which carry a larger proportion of canonical three-tetrad quadruplex forming sequences (59). Two-tetrad structures are usually less stable than three-tetrad G4 DNA. In general terms, there may be less of a G4 barrier to replication in *E. coli*, *Saccharomyces* and *Arabidopsis* compared to mammalian genomes, which could have influenced the evolution of specialized replication factors involved in copying these non-canonical template structures. Of course, the factors driving the natural selection of Rev1 isoforms with insert-2 and possessing enhanced G4 binding affinity were undoubtedly very complex. An argument against the view that the addition of insert-2 to Rev1 corresponded with the evolution of a more stable G4 landscape may be found in the fact that Rev1 from *Leishmania donovani* and *Trypanosoma cruzi* both lack insert-2 (Supplementary Figure S10), yet, the fraction of G4 motifs predicted have high stability in the *L. donovani* genome is similar to that observed for the human and mouse genomes (59). It seems more plausible that enrichment of stable G4 motifs observed in specific promoters could have helped drive selection for a more robust set of G4 resolving activities in humans and perhaps some other vertebrates. The strong association of high-stability quadruplexes in the regulatory regions of human and mouse genes related to cancer, as well as genes involved in development, neurological activity, and cardiac function, would seem to support such an idea (59).

Another potential clue to understanding the evolution of Rev1 may be held in analysis of how G4 structures populate the genomic landscape of cancer cells. Of the ∼700,000 G4 motifs found in the human genome, only a handful (∼10^3^) were found to exist in a form recognized by the G4-specific antibody BG4 when normal human epidermal keratinocytes were interrogated (30). This contrasts greatly with the tens of thousands of G4 structures identified in highly transcribed, nucleosome-depleted regions of the immortalized counterparts to the normal keratinocytes, mirroring increased G4 detection in cancer tissues compared to matched normal tissue (30,60). The number of G4 structures was even higher in patient-derived tumor xenograft (PDTX) models of breast cancer (BrCa), and the specific G4 enrichment patterns revealed distinct transcriptional programs for different BrCa subtypes (61). Related to the proposed function of Rev1, G4 sites in BrCa PDTX models were enriched in the number of somatic mutations, single nucleotide variants and copy number aberrations (61). Suppression of mutations in G4-rich regions of the genome through accurate and uninterrupted replication programs would have presumably provided a survival advantage for multi-cellular organisms by limiting deregulation of developmental genes and genes related to tumorigenesis. The evolution of robust G4-related activities for Rev1 – including features related to insert-2 and other residues involved in sequestering the ejected template base—could have aided in this regard.

## DATA AVAILABILITY

All data are available upon request.

## Supplementary Material

gkab041_Supplemental_File
