# Supplementary material for: Human Rev1 relies on insert-2 to promote selective binding and accurate replication of stabilized G-quadruplex motifs
Source: Nucleic Acids Res. 2021 Feb 8;49(4):2065–84. doi: 10.1093/nar/gkab041 (PMC7913688; doi:10.1093/nar/gkab041)
Supplement: gkab041_Supplemental_File [file gkab041_supplemental_file.pdf]

Supplementary materials for

Human Rev1 relies on insert-2 to promote selective binding and accurate replication of stabilized G-quadruplex motifs

Amit Ketkar[1], Lane Smith[1], Callie Johnson[3], Alyssa Richey[3], Makayla Berry[1], Jessica H. Hartman[4], Leena Maddukuri[1], Megan R. Reed[1], Julie Gunderson[5], Justin W.C. Leung[2], and Robert L. Eoff[1,]*

[1]Department of Biochemistry and Molecular Biology and [2]Department of Radiation Oncology, University of Arkansas for Medical Sciences, Little Rock, AR 72205, USA, [3]Arkansas School for Mathematics, Sciences, and the Arts, Hot Springs, AR 71901, USA, [4]Department of Biochemistry and Molecular Biology, Medical University of South Carolina, Charleston, SC 29425, USA, [5]Department of Physics, Hendrix College, Conway, AR 72032, USA.

* To whom correspondence should be addressed. Tel: +1 501 686 8343; Fax: +1 501 686 8169; Email: RLEoff@uams.edu

Running title: Human Rev1 interaction with G-quadruplex DNA

SUPPORTING INFORMATION

CONTENTS

Supplementary materials and methods

Table S1. CD melting temperatures of the ss-DNA G4 oligos used in this study.

Table S2. Equilibrium dissociation constants for hRev1$^{330-833}$ binding to ss-G4 and non-G4 DNA substrates in a buffer containing 100 mM LiCl.

Table S3. Equilibrium dissociation constants for hRev1$^{330-833}$ binding to ds-G4 and non-G4 DNA substrates in a buffer containing 100 mM KCl.

Table S4. hRev1$^{1-1251}$-derived peptides (trypsin-digested) identified by mass spectrometry.

Figure S1. Circular dichroism spectra of the DNA oligonucleotides used in this study.

Figure S2. Purification and CD spectra of hRev1$^{330-833}$ wild-type and mutant proteins.

Figure S3. Cloning, purification, DNA binding and enzyme activity of full-length hRev1$^{1-1251}$.

Figure S4. Schematic showing the DNA sequence of the pSP189 plasmids used in the forward mutagenesis assay.

Figure S5. Uncropped immunoblots of whole-cell lysates from HAP-1 *REV1*^{KO} cells transiently transfected with the pMH-SFB-Rev1 wild-type and muitant plasmids.

Figure S6. Alignment of the G4-*supF* plasmid sequences obtained from white MBM7070 *E. coli* colonies after replication in HAP-1 cells.

Figure S7. Alignment of the G4-*supF* plasmid sequences obtained from white MBM7070 *E. coli* colonies after replication in HAP-1 *REV1*^{KO} cells.

Figure S8. Sequence information, circular dichroism spectra and DNA binding of the putative G4-forming sequence identified at the *REV1* promoter.

Figure S9. Binding affinity of hRev1$^{330-833}$ to primer-template G4 DNA substrates.

Figure S10. Alignment of the Rev1 polymerase domain from multiple species.

Supplementary materials and methods

Circulr dichroism spectroscopy -

Circular dichroism (CD) spectroscopy for all DNA oligonucleotide substrates was performed on a Jasco J-1100 spectropolarimeter (Jasco, Easton, MD, USA). The ss-DNA G4 or non G4 oligos, or the corresponding primer-template ds-DNA substrates were prepared by annealing, as described in Methods. Solutions were prepared in 10 mM Tris-HCl, pH 7.5, containing either 100 mM KCl or LiCl. For measuring spectra, DNA was taken at a concentration of 5 µM in a total volume of 200 µL of buffer in a quartz cuvette with pathlength of 1 mm. Spectra were measured between the wavelength range of 230 nm to 330 nm, with a band-width of 1 nm, data pitch of 0.1 nm, at a scanning speed of 100 nm/min. Spectra were reported as an average of three scans. A similar scan of the buffer alone was used as blank to subtract and obtain the corrected spectra for oligo substrates. Thermal stability for the ss-G4 oligo substrates was measured by monitoring the change in CD signal at the corresponding peak wavelength, across a temperature range of 4°C to 95°C, by applying a melting temperature gradient of 1°C/min. An average of two scans was reported. Data analysis was performed using Graphpad Prism (San Diego, CA, USA), and the melting temperature values were obtained by fitting the data to a four-parameter logistic model allowing a variable slope.

Immunoblotting -

Aliquots at different stages during purification (lysate, supernatant after interaction with streptavidin-Sepharose beads, biotin-eluted fraction, etc.) from the large-scale culture of HEK293T cells stably expressing SFB-tagged hRev1$^{1\text{-}1251}$ were retained during protein purification. These were then loaded on a 4−20% gradient SDS-PAGE gel (Bio-Rad Laboratories, Hercules, CA, USA), and electrophoresis was performed at 120 V for 80 min. The separated proteins were transferred to a 0.2 µm polyvinylidene difluoride membrane (Bio-Rad; Cat # 162-0177) at 4°C at 200 mA for 90 min. After blocking with 5% (w/v) nonfat milk in 1X Tris-buffered saline (TBS), western blotting to detect the presence of SFB-tagged hRev1$^{1\text{-}1251}$ was performed by probing the membrane with the rabbit polyclonal anti-FLAG epitope antibody (Novus Biologicals; Cat # NB600-345). Dilution of the primary antibody was 1:1000 made in 1X TBS containing 1% (w/v) BSA. The blots were also probed with a rabbit anti-GAPDH primary antibody (Cell Signaling Technologies, Danvers, MA, USA; Cat #2118S) used at 1:10,000 dilution as loading control. Horseradish peroxidase-coupled anti-rabbit secondary antibody (Thermo-Scientific; Cat #32460) was used at a 1:2000 dilution. Blot was developed by enhanced chemiluminescence using the ECL kit (Bio-Rad; Cat # 170-5060) and the bands were visualized on a ChemiDoc MP imaging system (Bio-Rad; Cat # 12003154).

For the HAP-1 *REV1*^{KO} cells transiently transfected to express the SFB-tagged hRev1$^{1\text{-}1251}$ wild-type or mutant proteins, whole cell lysates were prepared 48 hrs post-transfection. *REV1*^{KO} cells that were sham-transfected (no plasmid) were used as untransfected control. Protein concentration of the lysates was estimated using the Pierce BCA assay kit (Thermo-Fisher Cat#23225). 50 µg of each lysate sample was loaded and separated by gel electrophoresis followed by transfer and blocking as described earlier. The immunoblot was then probed with the rabbit polyclonal anti-FLAG epitope antibody (Novus Biologicals; Cat # NB600-345; 1:1000 [v/v] dilution) to detect the level of each SFB-

hRev1 protein expression. The blot was subsequently also probed for loading control, with a rhodamine-conjugated anti-actin FAB (Bio-Rad12004164; 1:2000 [v/v] dilution).

Table S1. Melting temperatures of the G4 ssDNA substrates used in the study, measured by CD, in 10 mM Tr-s-Cl buffer (pH 7.5) containing 100 mM KCl/LiCl.

	**T_m [a](°C) [100 mM KCl]**	**T_m (°C) [100 mM LiCl]**
**Myc 14/23**	91 ± 3	52 ± 4
**Myc 2/11**	84 ± 4	49 ± 2
**Rev1-prom**	80 ± 3	51 ± 4
**Bcl-2 1245**	66 ± 5	53 ± 3
**KRAS 22RT**	56 ± 3	44 ± 1
**TBA**	48 ± 7	38 ± 4
**hTelo-4**	62 ± 5	41 ± 4

[a] T_m values reported represent the mean ± SD (n=3).

Table S2. Equilibrium dissociation constants for $hRev1^{330-833}$ binding to ss-G4 and non-G4 DNA substrates in a buffer containing 100 mM LiCl.[b]

	$K_{D.DNA}$		Fold preference for G4 DNA
	**Non-G4 (nM)**	**G4 (nM)**	($K_{D,NonG4\ DNA}/K_{D,G4\ DNA}$)
**Myc 14/23**	760 ± 290	220 ± 70	4
**Myc 2/11**	-	90 ± 20	8
**Rev1-prom**	260 ± 120	180 ± 70	2
**Bcl-2 1245**	750 ± 340	200 ± 40	4
**KRAS 22RT**	1620 ± 800	250 ±130	7
**TBA**	310 ± 40	140 ± 20	2
**hTelo-4**	920 ± 120	630 ± 100	2

[b]Fluorescence polarization experiments were performed by titrating $hRev1^{330-833}$ into a solution containing the indicated ss-DNA substrate containing 100 mM LiCl. The resulting equilibrium dissociation constant values were calculated by fitting the resulting polarization values to a quadratic equation. Data represent the mean ± SD (n=3).

Table S3. Equilibrium dissociation constants for hRev1$^{330-833}$ binding to ds-G4 and non-G4 DNA substrates in a buffer containing 100 mM KCl.[c]

	$K_{D.DNA}$		Fold preference for G4 DNA
	Non-G4 (nM)	G4 (nM)	($K_{D,NonG4\ DNA}/K_{D,G4\ DNA}$)
Myc 14/23	770 ± 80	59 ± 9	13
Myc 2/11	-	45 ± 6	17
Rev1-prom	154 ± 30	11 ± 2	14
Bcl-2 1245	660 ± 120	97 ± 20	7
KRAS 22RT	1120 ± 80	120 ±10	9
TBA	270 ± 30	165 ± 25	2
hTelo-4	1600 ± 320	840 ± 50	2

[c]Fluorescence polarization experiments were performed by titrating hRev1$^{330-833}$ into a solution containing the indicated ds-DNA substrate containing 100 mM KCl. The resulting equilibrium dissociation constant values were calculated by fitting the resulting polarization values to a quadratic equation. Data represent the mean ± SD (n=3).

Table S4. hRev1$^{1\text{-}1251}$-derived peptides (trypsin-digested) identified by mass spectrometry.

Peptide Sequence (Trypsin cleavage sites in parentheses)	m/z	M.W.	Charge state	Residue (Start-End)
(K)AAPSVPSKPSDcNFISNFYSHSR(L)*	856.73	2567.18	+3	335-357
(R)LHHISMWK(C)	526.28	1050.54	+2	358-365
(K)cELTEFVNTLQR(Q)	755.37	1508.73	+2	366-377
(R)QSnGIFPGREK(L)*	617.31	1232.62	+2	378-388
(R)QSnGIFPGREK(L)**	683.33	1364.64	+2	378-388
(K)mKTGRSALVVTDTGDmSVLNSPR(H)*	650.57	2598.24	+4	392-414
(R)SALVVTDTGDMSVLNSPR(H)	621.32	1860.93	+3	397-414
(R)HQScImHVDMDcFFVSVGIR(N)	818.70	2453.07	+3	415-434
(R)NRPDLKGKPVAVTSNR(G)	438.75	1750.98	+4	435-450
(R)NRPDLKGKPVAVTSNR(G)	471.76	1883.00	+4	435-450
(K)GKPVAVTSNRGTGR(A)	700.39	1398.77	+2	441-454
(K)GKPVAVTSNRGTGR(A)	511.27	1530.79	+3	441-454
(R)GTGRAPLRPGANPQLEWQYYQNK(I)	661.84	2643.33	+4	451-473
(R)GTGRAPLRPGANPQLEWQYYQNK(I)	694.85	2775.35	+4	451-473
(R)APLRPGANPQLEWQYYQNK(I)	758.39	2272.14	+3	455-473
(K)GKAADIPDSSLWENPDSAQAnGIDSVLSR(A)	1005.48	3013.43	+3	477-505
(K)AADIPDSSLWENPDSAQANGIDSVLSRAEIAScSYEAR(Q)	1355.96	4064.86	+3	479-516
(R)AEIAScSYEARQLGIK(N)	599.31	1794.89	+3	506-521
(R)AEIAScSYEARQLGIK(N)	643.31	1926.92	+3	506-521
(R)QLGIKnGMFFGHAK(Q)	516.94	1547.79	+3	517-530
(K)NGMFFGHAK(Q)	504.74	1007.46	+2	522-530
(K)QLcPNLQAVPYDFHAYK(E)	1032.50	2062.99	+2	531-547
(K)LTPDEFANAVR(M)	616.82	1231.62	+2	585-595
(R)MEIKDQTK(C)	496.76	991.50	+2	596-603
(K)cAASVGIGSNILLAR(M)	751.41	1500.81	+2	604-618
(K)cAASVGIGSNILLARmATR(K)	703.69	2108.05	+3	604-622
(R)KAKPDGQYHLKPEEVDDFIR(G)	597.06	2384.21	+4	623-642
(K)AKPDGQYHLKPEEVDDFIR(G)	565.04	2256.12	+4	624-642
(R)GQLVTNLPGVGHSmESK(L)	885.45	1768.88	+2	643-659
(R)GQLVTNLPGVGHSmESKLASLGIK(T)	818.11	2451.32	+3	643-666
(K)LASLGIKTcGDLQYMTmAK(L)	706.35	2116.04	+3	660-678
(K)TcGDLQYmTMAK(L)	717.81	1433.60	+2	667-678
(K)LQKEFGPKTGQmLYR(F)	453.74	1810.94	+4	679-686
(K)TGQmLYRFcR(G)	493.89	1478.64	+3	687-696
(K)TGQMLYRFcR(G)	488.56	1462.65	+3	687-696
(R)GLDDRPVRTEK(E)	709.36	1416.70	+2	697-707
(R)GLDDRPVRTEKER(K)	785.92	1569.82	+2	697-709
(K)ERKSVSAEINYGIR(F)	541.29	1620.86	+3	708-721
(R)KSVSAEINYGIR(F)	446.25	1335.71	+3	710-721
(K)SVSAEINYGIR(F)	403.55	1207.62	+3	711-721

(R)FTQPKEAEAFLLSLSEEIQR(R)	779.41	2335.21	+3	722-741
(K)EAEAFLLSLSEEIQR(R)	867.95	1733.88	+2	727-741
(R)RLEATGmKGK(R)	553.80	1105.59	+2	742-751
(R)LTLKImVRKPGAPVETAK(F)	492.80	1967.16	+4	753-770
(K)ImVRKPGAPVETAK(F)	548.96	1643.87	+3	757-770
(K)ImVRKPGAPVETAK(F)	756.93	1511.85	+2	757-770
(R)KPGAPVETAKFGGHGIcDNIAR(T)	574.55	2294.16	+4	761-782
(K)FGGHGIcDNIARTVTLDQATDNAK(I)	677.32	2705.25	+4	771-794
(R)TVTLDqATDNAK(I)	639.31	1276.61	+2	783-794
(R)TVTLDQATDnAKIIGK(A)	563.64	1687.90	+3	783-798
(K)IIGKAmLNMFHTMK(L)	550.96	1649.85	+3	795-808
(K)AMLNmFHTmKLNISDMR(G)	695.66	2083.97	+3	799-815

* *Lowercase 'n' indicates deamidation (+1) of Asn residues, while lowercase 'c' indicates carbamidomethyl modification (+57), and 'm' indicates oxidation (+16) of Met residues.*

** *Arg residues highlighted in red were modified by HPG (+132).*

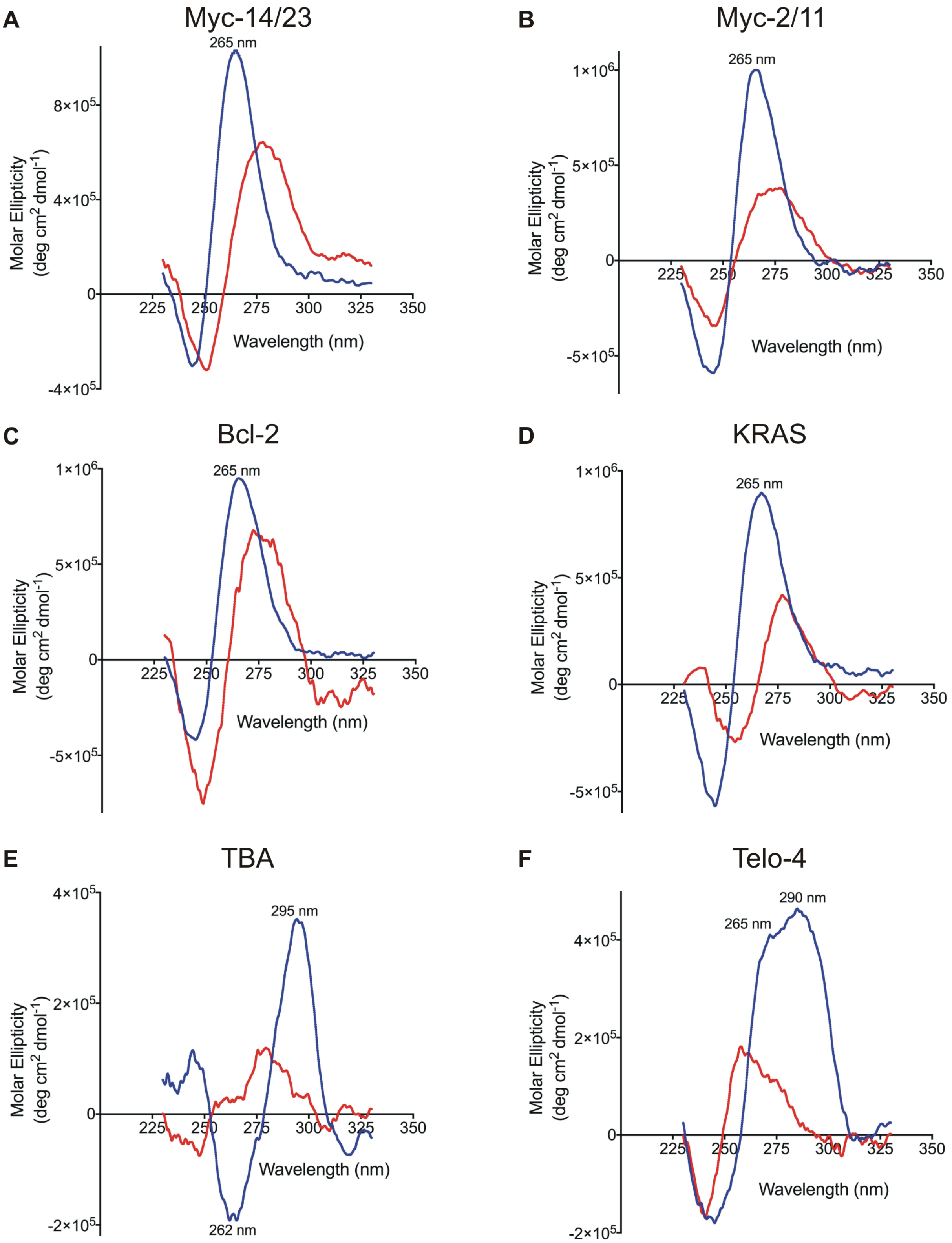

Figure S1. Circular dichroism spectra of the ss-DNA oligonucleotides used in this study. Spectra measured in 100 mM KCl are shown in *blue*, while those in 100 mM LiCl are shown in *red*. Positions of signature peaks and troughs for parallel (265 nm), anti-parallel (262 nm and 295 nm) and mixed (265 nm and 290 nm) G4-DNA CD curves are marked.

A

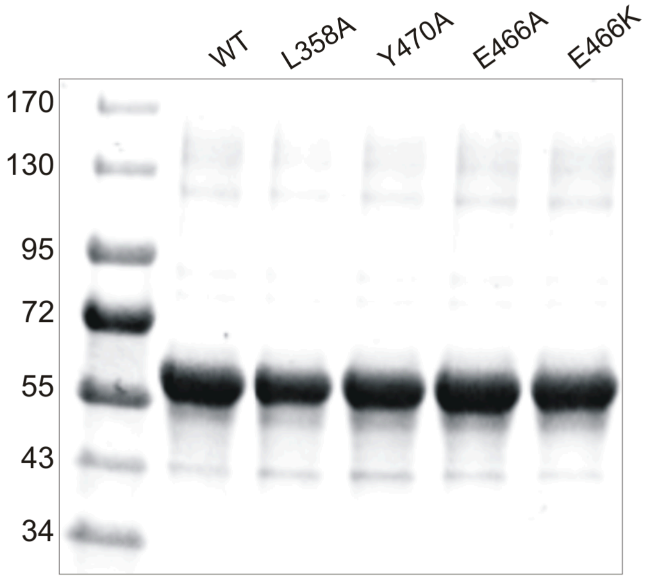

B

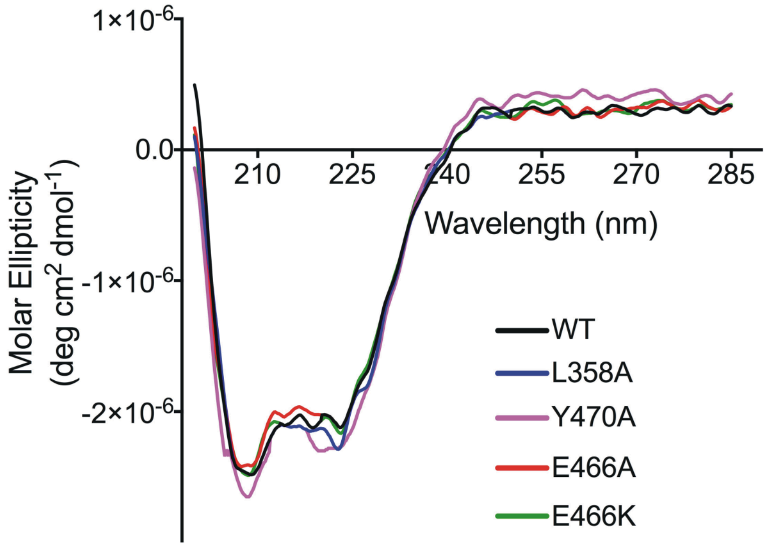

Figure S2. hRev1$^{330\text{-}833}$ wild-type and mutant proteins were overexpressed in *E. coli* BL21 (DE3) cells and purified. (**A**) All the hRev1$^{330\text{-}833}$ proteins were purified to homogeneity. A Coomassie brilliant blue R-250 stained SDS-PAGE gel with 10 μg of each protein is shown. (**B**) CD spectra of the purified hRev1$^{330\text{-}833}$ proteins are shown. The spectra of all proteins overlapped, indicative of similar folding for the wild-type and mutant proteins.

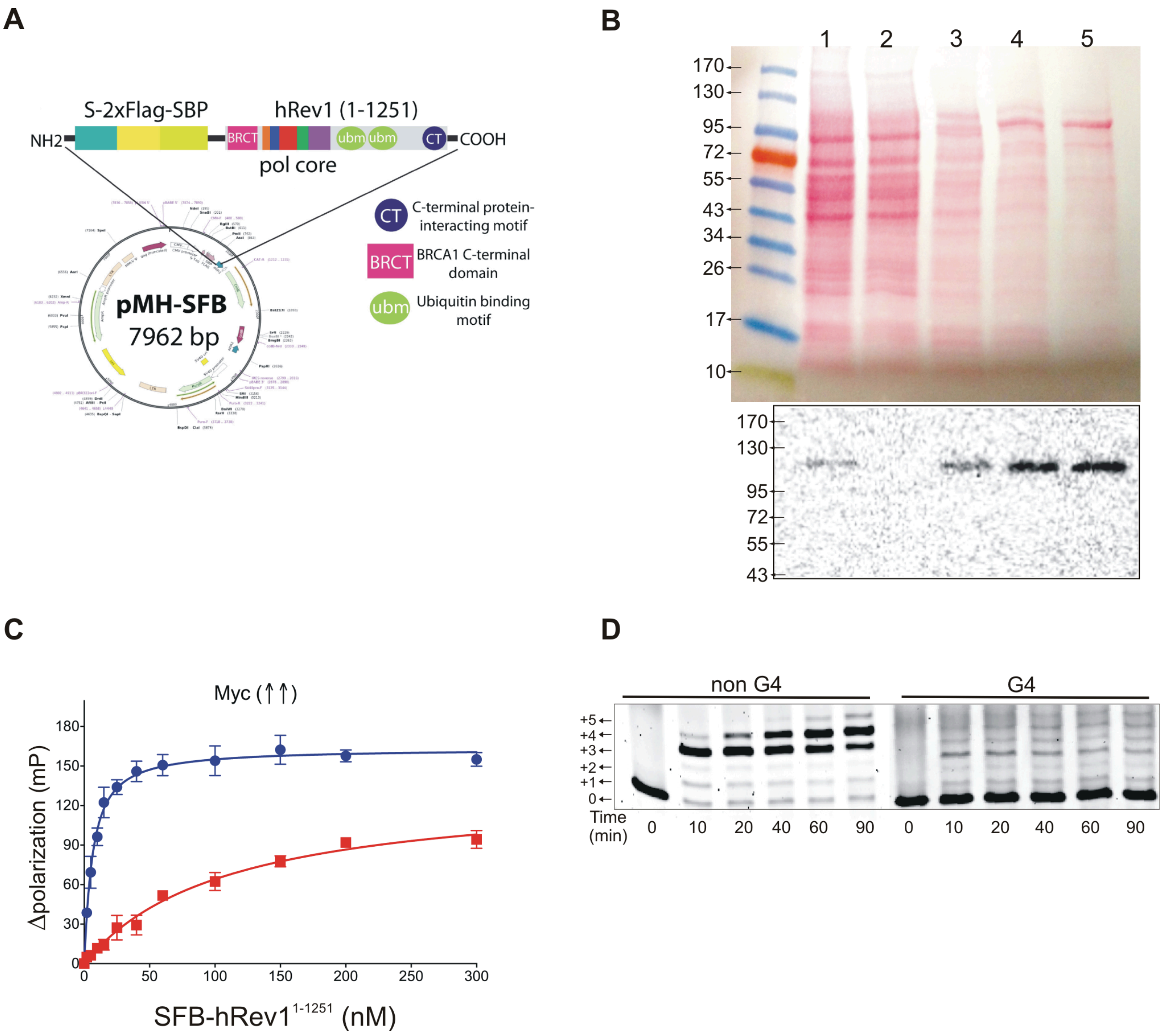

Figure S3. Full-length hRev1 was used in *supF*-forward mutagenesis assay and biochemical experiments. (**A**) Full-length hRev1 (a.a. 1-1251) was cloned into the Gateway-compatible pMH-SFB mammalian expression vector as a N-terminal tandem S protein-FLAG-streptavidin-Binding peptide (SFB) tagged construct. The domain organization of the construct is shown, where the affinity tag and hRev1 domains are labeled. *S*, S-protein; *2xFLAG*, two tandem FLAG-peptide sequences; *SBP*, streptavidin-binding peptide; *BRCT*, BRCA1 C terminus domain; *pol core*, hRev1 polymerase core domain (a.a. 330-833) colored according to the scheme identical to that used in **Figure 2C** to show the sub-domains; *ubm*, ubiquitin-binding motif; *CT*, C-terminal domain (a.a. 1150-1251). This plasmid was used to transfect HAP-1 mammalian cells for the forward mutagenesis assay. All the mutant hRev1 constructs described in the mutagenic assay were subsequently made using this construct. (**B**) HEK293T cells stably expressing SFB-tagged hRev1$^{1\text{-}1251}$ were generated using the construct described in **A**, and the overexpressed protein was purified using affinity chromatography with

streptavidin-sepharose beads. Aliquots from different stages of the protein purification protocol were separated using SDS-PAGE gel electrophoresis. *Lane 1*, Lysate from the HEK293T cells; *lane 2*, supernatant after interaction with streptavidin-sepharose beads; *lane 3*, first eluate with 2 mg/mL biotin; *lane 4*, biotin eluate after second round of binding; *lane 5*, final dialyzed sample. A major band at ~120 kDa was observed for the purified protein. Molecular weight markers are shown in the far-left lane. The panel below the Ponceau-stained blot shows the western blot probed with an anti-FLAG antibody. A single band was detected for SFB-hRev1 at different stages of purification. (**C**) DNA binding properties of the purified hRev1$^{1\text{-}1251}$ protein were studied using the Myc 14/23 substrate. Binding curves for the Myc 14/23 G4 substrate (*blue*) and the non-G4 control (*red*) are shown. The values for the equilibrium dissociation constants are given in **Table 2**. (**D**) Enzyme activity on both G4- and non-G4 template-primer substrates was tested for the purified hRev1$^{1\text{-}1251}$ protein, in an assay as described in Methods and **Figure 4**.

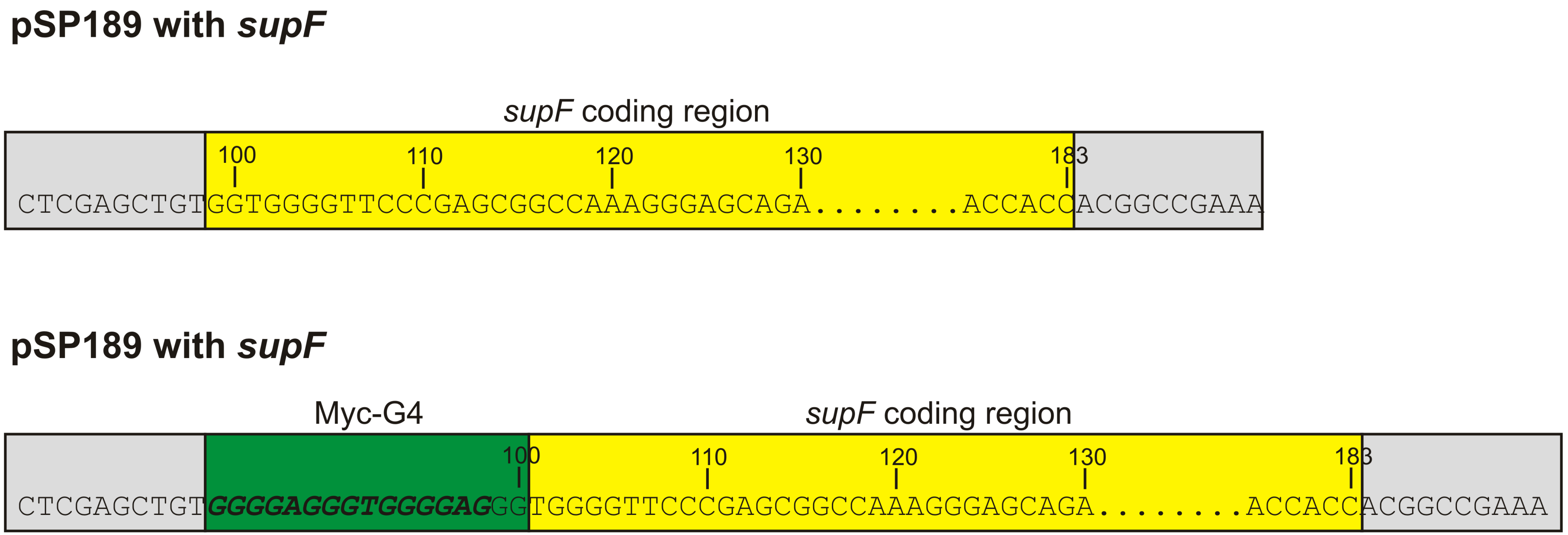

Figure S4. Schematic showing the DNA sequence of the pSP189 plasmids used in the forward mutagenesis assay. The unmodified pSP189 plasmid sequence is shown in the top panel, with the *supF*-tRNA coding region marked in *yellow*. The numbering is according to Seidman *et al* (1). In the lower panel, the region marked in *green* indicates the Myc-G4 sequence inserted into the pSP189 plasmid. The color scheme is identical to that used in **Figure 6**.

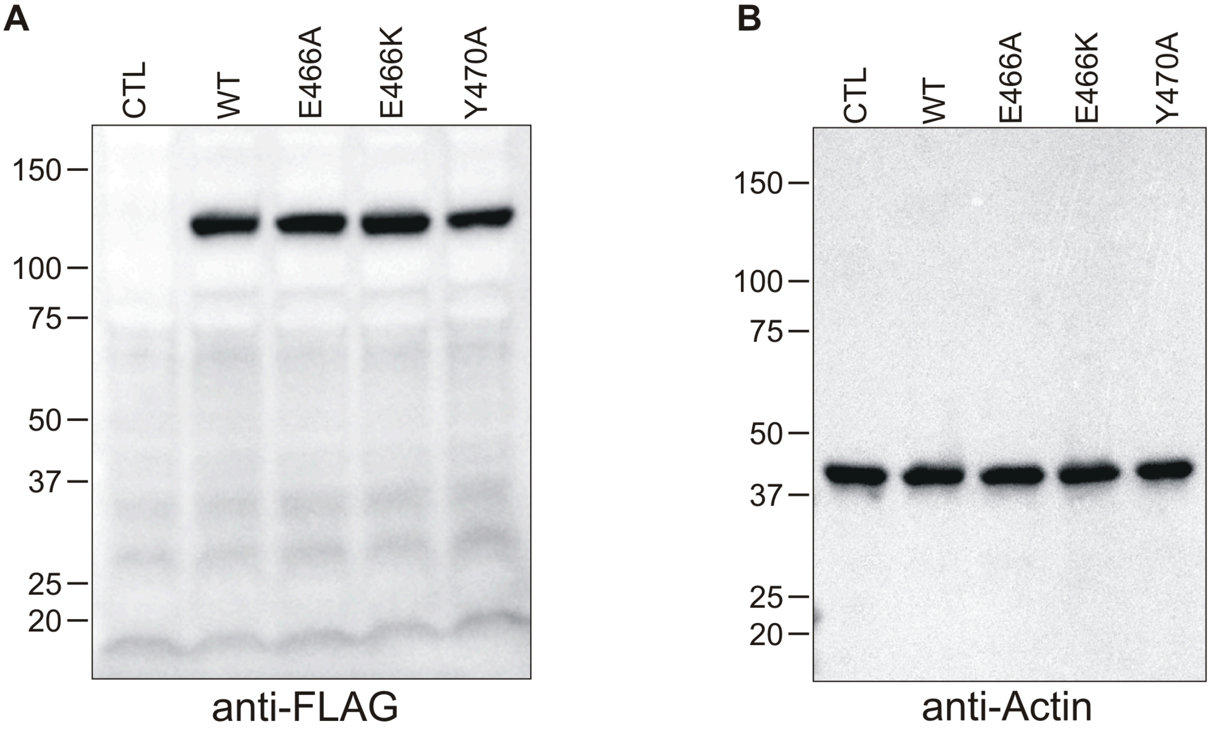

Figure S5. Uncropped Western immunoblots of whole-cell lysates from HAP-1 *REV1*^{KO} cells transiently transfected with the pMH-SFB-Rev1 plasmids. The HAP-1 *REV1*^{KO} cells were transiently transfected with different constructs of the pMH-SFB-Rev1 plasmid, expressing the wild-type, or mutant form of hRev1, and these were used in the complementation experiments in the supF forward mutagenesis assay. Cropped versions of these blots are shown in Figure 5A. **(A)** The immunoblot was probed with a rabbit anti-FLAG tag polyclonal primary antibody (Novus Biologicals Cat# NB600-345; 1:1000 [v/v] dilution), to detect the level of protein expression for each of the SFB-tagged hRev1 proteins as indicated above each lane. **(B)** The immunoblot described in A was probed with a rhodamine-conjugated anti-actin loading control antibody FAB (Bio-Rad Cat#12004164; 1:2000 [v/v] dilution.

HAP-1 No PDS

```
G4_supF      GAATTCGAGAGCCCTGCTCGAGCTGTGGGGAGGGTGGGGAGGGTGGGGTTCCCGAGCGGCCAAAGGGAGCAGACTCTAAATCTGCCGTCATCGAC
No-PDS_1     GAATTCGAGAGCCCTGCTCGAGCTGTGGGGAGGGTAGGGAGGATGGGGTTCCCGAGCGGTCAAAGGTAGCAGACTCTAAATCTGCCGTCATCGAC
No-PDS_2     GAATTCGAGAGCCCTGCTCGAGCTGTGCGGAGGGTGGGGAGGGTGGGGTTCCCTAGCGGCCA-AGTGAGGAGACTCTAAATCTGCCGTCATCGAC
No-PDS_3     GAATTCGAGAGCCCTGCTCGAGCTGTGTGGAGGGTGGGGAGGGTGGGGTTCCCGAGCGGCCAAAGTTAGCAGACTCTAAATCTGCCGTCATCGAC
No-PDS_4     GAATTCGAGAGCCCTGCTCGAGCTGTGCGGAGGGTGGGGAGGGTGGGGTTCCCTAGCGGTCAAAGGTAGCAGACTCTAAATCTGCCGTCATCGAC
No-PDS_5     GAATTCGAGAGCCCTGCTCGAGCTGTGTGGAGGGTGGGGAGGGTGGGGTTCCCGAGCGGCCA-AGTGAGGAGACTCTAAATCTGCCGTCATCGAC
No-PDS_6     GAATTCGAGAGCCCTGCTCGAGCTGTGGGGAGGGTAGGGAGGATGGGGTTCCCGAGCGGCCAAAGTTAGCAGACTCTAAATCTGCCGTCATCGAC
No-PDS_7     GAATTCGAGAGCCCTGCTCGAGCTGTGCGGAGGGTGGGGAGGGTGGGGTTCCCTAGCGGCCA-AGTGAGGAGACTCTAAATCTGCCGTCATCGAC
No-PDS_8     GAATTCGAGAGCCCTGCTCGAGCTGTGTGGAGGGTGGGGAGGGTGGGGTTCCCGAGCGGCCAAAGTTAGCAGACTCTAAATCTGCCGTCATCGAC
No-PDS_9     GAATTCGAGAGCCCTGCTCGAGCTGTGCGGAGGGTGGGGAGGGTGGGGTTCCCTAGCGGTCAAAGGTAGCAGACTCTAAATCTGCCGTCATCGAC
No-PDS_10    GAATTCGAGAGCCCTGCTCGAGCTGTGTGGAGGGTGGGGAGGGTGGGGTTCCCGAGCGGCCA-AGTGAGGAGACTCTAAATCTGCCGTCATCGAC
             *************************** ******* ****** ********** ***** ** **  ** *************************

G4_supF      TTCGAAGGTTCGAATCCTTCCCCCACCACCACGGCCGAAATTCGGTACCCGGATCCTTAGC
No-PDS_1     TTCGGAGGTTCTAATCCTTCCCCCACCACCACGGCCGAAATTCGGTACCCGGATCCTTAGC
No-PDS_2     TTCGAAGATTCGATTCCTTCCCCCACGACCACGGCCGAAATTCTGTACCCGGATCCTTAGC
No-PDS_3     -TCGAAGGTTCGAATCCTTCCCCCACCACCACGGCCGAAATTCGGTACCCGGATCCTTAGC
No-PDS_4     TTCGGAGGTTCTAATCCTTCCCCCACGACCACGGCCGAAATTCTGTACCCGGATCCTTAGC
No-PDS_5     TTCGAAGATTCGATTCCTTCCCCCACGACCACGGCCGAAATTCTGTACCCGGATCCTTAGC
No-PDS_6     -TCGAAGGTTCGAATCCTTCCCCCACCACCACGGCCGAAATTCGGTACCCGGATCCTTAGC
No-PDS_7     TTCGAAGATTCGATTCCTTCCCCCACGACCACGGCCGAAATTCTGTACCCGGATCCTTAGC
No-PDS_8     -TCGAAGGTTCGAATCCTTCCCCCACCACCACGGCCGAAATTCGGTACCCGGATCCTTAGC
No-PDS_9     TTCGGAGGTTCTAATCCTTCCCCCACGACCACGGCCGAAATTCTGTACCCGGATCCTTAGC
No-PDS_10    TTCGAAGATTCGATTCCTTCCCCCACCACCACGGCCGAAATTCGGTACCCGGATCCTTAGC
              *** ** *** * ************ **************** *****************
```

HAP-1 0.5 μM PDS

```
G4_SupF        GAATTCGAGAGCCCTGCTCGAGCTGTGGGGAGGGTGGGGAGGGTGGGGTTCCCGAGCGGCCAAAGGGAGCAGACTCTAAATCTGCCGTCATCGAC
0.5-PDS_1      GAATTCGAGAGCCCTGCTCGAGCTGTGGGGAGGGTGGGAAGGGTGGGGT-CCCGATCGGCCAAAGGGATCAGACTCTA-ATCTGCCGTCATCGTC
0.5-PDS_2      GAATTCTAGAGCCCTGCTCGAGCTGTGGGGAGGCTGGGTAGGGTGGGGTTCCCGAGCGGCCAAAGTGAGAAGACTCTAAATCTGCCGTCATCGAC
0.5-PDS_3      GAATTCGATAGCCCTGCTCGAGCTGTGGGAAGGGTGGGGAGGGTGGGGTTCCCGAGCGGCCAAAGGTAGCAGACTCTAAATCTGC--TCATCGAC
0.5-PDS_4      GAATTCGAGAGCCCTGCTCGAGCTGTGGGGAGGGTGGGAAGGGTGGGGT-CCCGATCGGCCAAAGGGATCAGACTCTA-ATCTGCCGTCATCGTC
0.5-PDS_5      GAATTCTAGAGCCCTGCTCGAGCTGTGGGGAGGCTGGGTAGGGTGGGGTTCCCGAGCGGCCAAAGTGAGAAGACTCTAAATCTGCCGTCATCGAC
0.5-PDS_6      GAATTCGATAGCCCTGCTCGAGCTGTGGGAAGGGTGGGGAGGGTGGGGTTCCCGAGCGGCCAAAGGTAGCAGACTCTAAATCTGC--TCATCGAC
0.5-PDS_7      GAATTCGATAGCCCTGCTCGAGCTGTGGGAAGGGTGGGGAGGGTGGGGTTCCCGAGCGGCCAAAGGGATCAGACTCTA-ATCTGCCGTCATCGTC
0.5-PDS_8      GAATTCGAGAGCCCTGCTCGAGCTGTGGGGAGGGTGGGAAGGGTGGGG-TCCCGATCGGCCAAAGTGAGAAGACTCTAAATCTGCCGTCATCGAC
0.5-PDS_9      GAATTCTAGAGCCCTGCTCGAGCTGTGGGGAGGCTGGGTAGGGTGGGGTTCCCGAGCGGCCAAAGGTAGCAGACTCTAAATCTGC--TCATCGAC
0.5-PDS_10     GAATTCGATAGCCCTGCTCGAGCTGTGGGAAGGGTGGGGAGGGTGGGGTTCCCGAGCGGCCAAAGGGATCAGACTCTA-ATCTGCCGTCATCGTC
               ****** * ******************** *** **** *********  ***** *********  *  ******** ******  ****** *

G4_SupF        TTCGAAGGTTCGAATCCTTCCCCCACCACCACGGCCGAAATTCGGTACCCGGATCCTTAGC
0.5-PDS_1      TTCGAAGGTTCGAATCCTTCCCCCACCACCACGGCCGAAATTCGTTACCCGGATCCTTAGC
0.5-PDS_2      --CGAAGGTTCGAAACCTTCCCCCAT-ACCACGGCCGAAATTCGGTACCCGGATCCTTAGC
0.5-PDS_3      TTCGAAGGTTCGAATCCTTCCCCCACCTCCACGGCCGAAATTAGGTACCCGGATCCTTAGC
0.5-PDS_4      TTCGAAGGTTCGAATCCTTCCCCCACCACCACGGCCGAAATTCGTTACCCGGATCCTTAGC
0.5-PDS_5      --CGAAGGTTCGAAACCTTCCCCCAT-ACCACGGCCGAAATTCGGTACCCGGATCCTTAGC
0.5-PDS_6      TTCGAAGGTTCGAATCCTTCCCCCACCTCCACGGCCGAAATTAGGTACCCGGATCCTTAGC
0.5-PDS_7      TTCGAAGGTTCGAATCCTTCCCCCACCACCACGGCCGAAATTCGTTACCCGGATCCTTAGC
0.5-PDS_8      --CGAAGGTTCGAAACCTTCCCCCAT-ACCACGGCCGAAATTCGGTACCCGGATCCTTAGC
0.5-PDS_9      TTCGAAGGTTCGAATCCTTCCCCCACCACCACGGCCGAAATTCGTTACCCGGATCCTTAGC
0.5-PDS_10     TTCGAAGGTTCGAATCCTTCCCCCA-TACCACGGCCGAAATTCGGTACCCGGATCCTTAGC
                 ************ **********   ************** * ****************
```

Figure S6. Alignment of the G4-*supF* plasmid sequences obtained from white MBM7070 *E. coli* colonies. These plasmids were first allowed to replicate in HAP-1 cells grown in the presence or absence of 0.5 μM PDS. After extracting these plasmids from the HAP-1 cells, they were used to transform the bacterial indicator strain as described in Methods. The top sequence is for the parent G4-*supF* plasmid (before transfection into HAP-1 cells), while lower sequences were obtained from the white-colony transfectants. Ten colonies per condition were used for sequencing.

The positions of conserved/unmutated bases (in all the ten sequences) are marked by asterisks, while deletions are marked by dashes in each sequence. The shading/coloring of the unmodified G4-*supF* sequence marks the zones I to IV as mentioned in **Figure 6** of the manuscript.

HAP-1 *REV1*^{KO} No PDS

```
G4_SupF         GAATTCGAGAGCCCTGCTCGAGCTGTGGGGAGGGTGGGGAGGGTGGGGTTCCCGAGCGGCCAAAGGGAGCAGACTCTAAATCTGCCGTCATCGAC
No-PDS_1        CAATTCGAGAGCCCTGCTCGAGCTGTGAGGAGCGTGGGGAGGGTGGGGTTCCCGAACGGCCAAATGGAGCAGACTCTAAAACT-CCGTCATCGAC
No-PDS_2        CAATTCGAGAGCCCAGCTCGAGCTGTTGGGAGGGTGTGGAGGGTGGGGTTCC--AGCGGCCAAG---GTCAGACTCTAAATCT-GCCGTATCGAC
No-PDS_3        GAATTCTAGAG--CTGCTCGAGCTGTGGGGAGTGTGGGGAGGGTTTGGTTCCCGAGCGGCCAAAAGTAGCAGACTCTAAATCT--CGTCATCGAC
No-PDS_4        CAATTCGAGAGCCCTGCTCGAGCTGTGAGGAGCGTGGGGAGGGTGGGGTTCCCGAACGGCCAAGG---TCAGACTCTAAATCTGCCGT-ATCGAC
No-PDS_5        CAATTCGAGAGCCCAGCTCGAGCTGTTGGGAGGGTGTGGAGGGTGGGGTTCC--AGCGGCCAAATGGAGCAGACTCTAAAACT-CCGTCATCGAC
No-PDS_6        GAATTCTAGAG--CTGCTCGAGCTGTGGGGAGTGTGGGGAGGGTTTGGTTCCCGAGCGGCCAAGGT---CAGACTCTAAATCTGCCGT-ATCGAC
No-PDS_7        CAATTCGAGAGCCCAGCTCGAGCTGTTGGGAGGGTGTGGAGGGTGGGGTTCC--AGCGGCCAAAAGTAGCAGACTCTAAATCT-C-GTCATCGAC
No-PDS_8        GAATTCTAGAG--CTGCTCGAGCTGTGGGGAGTGTGGGGAGGGTTTGGTTCCCGAGCGGCCAAGGT---CAGACTCTAAATCTGCCGT-ATCGAC
No-PDS_9        CAATTCGAGAGCCCTGCTCGAGCTGTGAGGAGCGTGGGGAGGGTGGGGTTCCCGAACGGCCAAGG---TCAGACTCTAAATCTGCCGT-ATCGAC
No-PDS_10       CAATTCGAGAGCCCAGCTCGAGCTGTTGGGAGGGTGTGGAGGGTGGGGTTCC--AGCGGCCAAAAGTAGCAGACTCTAAATCT-C-GTCATCGAC
                  **** ****  * ***********  **** *** *******  ******  * *******      *********** **      ******

G4_SupF         TTCGAAGGTTCGAATCCTTCCCCCACCACCACGGCCGAAATTCGGTACCCGGATCCTTAGC
No-PDS_1        TT---AGGTTCGAATCCTTCCCCCATCACCACGGCCGAAATTCGGTACCCGAATCCTTAGC
No-PDS_2        TTCG-AGGTTCGAATCCTTCACCCACCACCACGGCCGAAATTCAGTACCCGGATCCTTAGC
No-PDS_3        TTCGTAGGTTCGAATCTTTCCCCCACTACCACGGCCGAAATTCGATACCCGGATCCTTA--
No-PDS_4        TTCG-AGGTTCGAATCCTTCACCCATCACCACGGCCGAAATTCGGTACCCGAATCCTTAGC
No-PDS_5        TT---AGGTTCGAATCCTTCCCCCACCACCACGGCCGAAATTCAGTACCCGGATCCTTAGC
No-PDS_6        TTCG-AGGTTCGAATCCTTCACCCACTACCACGGCCGAAATTCGATACCCGGATCCTTA--
No-PDS_7        TTCGTAGGTTCGAATCTTTCCCCCACCACCACGGCCGAAATTCAGTACCCGGATCCTTAGC
No-PDS_8        TTCG-AGGTTCGAATCCTTCACCCACTACCACGGCCGAAATTCGATACCCGGATCCTTA--
No-PDS_9        TTCG-AGGTTCGAATCCTTCACCCATCACCACGGCCGAAATTCGGTACCCGAATCCTTAGC
No-PDS_10       TTCGTAGGTTCGAATCTTTCCCCCACCACCACGGCCGAAATTCAGTACCCGGATCCTTAGC
                **   *********** *** ****  ****************  *************
```

HAP-1 *REV1*^{KO} 0.5 μM PDS

```
G4_SupF         GAATTCGAGAGCCCTGCTCGAGCTGTGGGGAGGGTGGGGAGGGTGGGGTTCCCGAGCGGCCAAAGGGAGCAGACTCTAAATCTGCCGTCATCGAC
0.5-PDS_1       GAA-TCGAGAGCCCTGCTCGAGCTGTGGAGAGGGTGGGGAGGGTGGAGTCCCAA---GGCCATG----GAGGACTC--AATCTGTCGTCTTCGAC
0.5-PDS_2       AAATTCTAGAGCCCCGCTCGAGCTGTGAGGAGTGTGGGGAGGGTGACGTTCCTGAGCGGC----TGGAGCAGACTCTAAATCTGCCGTCATCGAC
0.5-PDS_3       AAATTCGAGAGCCCTGCTCGAGCTGTAGGGAGGGTGGGGAGGGTAGCGTTCCTGAGCGGCCAAATGGAGCAGACTCTAAACCTGCCGTC---GAC
0.5-PDS_4       GAA-TCGAGAGCCCTGCTCGAGCTGTGGAGAGGGTGGGGAGGGTGGAGTCCCAA---GGCCATG----GAGGACTC--AATCTGTCGTCTTCGAC
0.5-PDS_5       AAATTCTAGAGCCCCGCTCGAGCTGTGAGGAGTGTGGGGAGGGTGACGTTCCTGAGCGGC----TGGAGCAGACTCTAAATCTGCCGTCATCGAC
0.5-PDS_6       AAATTCGAGAGCCCTGCTCGAGCTGTAGGGAGGGTGGGGAGGGTAGCGTTCCTGAGCGGCCATG----GAGGACTC--AATCTGTCGTCTTCGAC
0.5-PDS_7       AAATTCGAGAGCCCTGCTCGAGCTGTAGGGAGGGTGGGGAGGGTAGCGTTCCTGAGCGGC----TGGAGCAGACTCTAAATCTGCCGTCATCGAC
0.5-PDS_8       GAA-TCGAGAGCCCTGCTCGAGCTGTGGAGAGGGTGGGGAGGGTGGAGTCCCAA---GGCCAAATGGAGCAGACTCTAAACCTGCCG---TCGAC
0.5-PDS_9       AAATTCTAGAGCCCCGCTCGAGCTGTGAGGAGTGTGGGGAGGGTGACGTTCCTGAGCGGCCATG----GAGGACTC--AATCTGTCGTCTTCGAC
0.5-PDS_10      AAATTCGAGAGCCCTGCTCGAGCTGTAGGGAGGGTGGGGAGGGTAGCGTTCCTGAGCGGC----TGGAGCAGACTCTAAATCTGCCGTCATCGAC
                 ** ** ******* ***********   *** ***********   ** **   ***        *  *****    ** *** **     ***

G4_SupF         TTCGAAGGTTCGAATCCTTCCCCCACCACCACGGCCGAAATTCGGTACCCGGATCCTTAGC
0.5-PDS_1       TTAA--GGTTT--T--CT---TCCCCCACTACGGCCGA-ATTCGATACCTGAATCCTTAGC
0.5-PDS_2       TTCGTAGGTTA--ATCCTTCCC--ACCACTACGGCCGA-ATTCGGAACCCGGATCCTTAGC
0.5-PDS_3       TTCG--GGTTC--ATCCTTCCCCA--ACTACGGC-----TTCGGTACCCGTATCCTTAGC
0.5-PDS_4       TTAA--GGTTT--T--CTTCCCCCACCACTACGGCCGA-ATTCGGAACCCGGATCCTTAGC
0.5-PDS_5       TTCGTAGGTTA--ATCCTTCCC----AACTACGGC-----TTCGGTACCCGTATCCTTAGC
0.5-PDS_6       TTAA--GGTTT--T--CTTCCCCCACCACTACGGCCGA-ATTCGGAACCCGGATCCTTAGC
0.5-PDS_7       TTCGTAGGTTA--ATCCTTCC--CA--ACTACGGC-----TTCGGTACCCGTATCCTTAGC
0.5-PDS_8       TTCG--GGTTC--ATCCT---TCCCCCACTACGGCCGA-ATTCGATACCTGAATCCTTAGC
0.5-PDS_9       TTAA--GGTTT--T--CTTCCCCCACCACTACGGCCGA-ATTCGGAACCCGGATCCTTAGC
0.5-PDS_10      TTCGTAGGTTA--ATCCTTCC--CA--ACTACGGC-----TTCGGTACCCGTATCCTTAGC
                **    ****      **         ** *****     ****  *** * *********
```

Figure S7. Alignment of the G4-*supF* plasmid sequences obtained from white MBM7070 *E. coli* colonies. These plasmids were first allowed to replicate in HAP-1 *REV1*^{KO} cells grown in the presence or absence of 0.5 μM PDS.

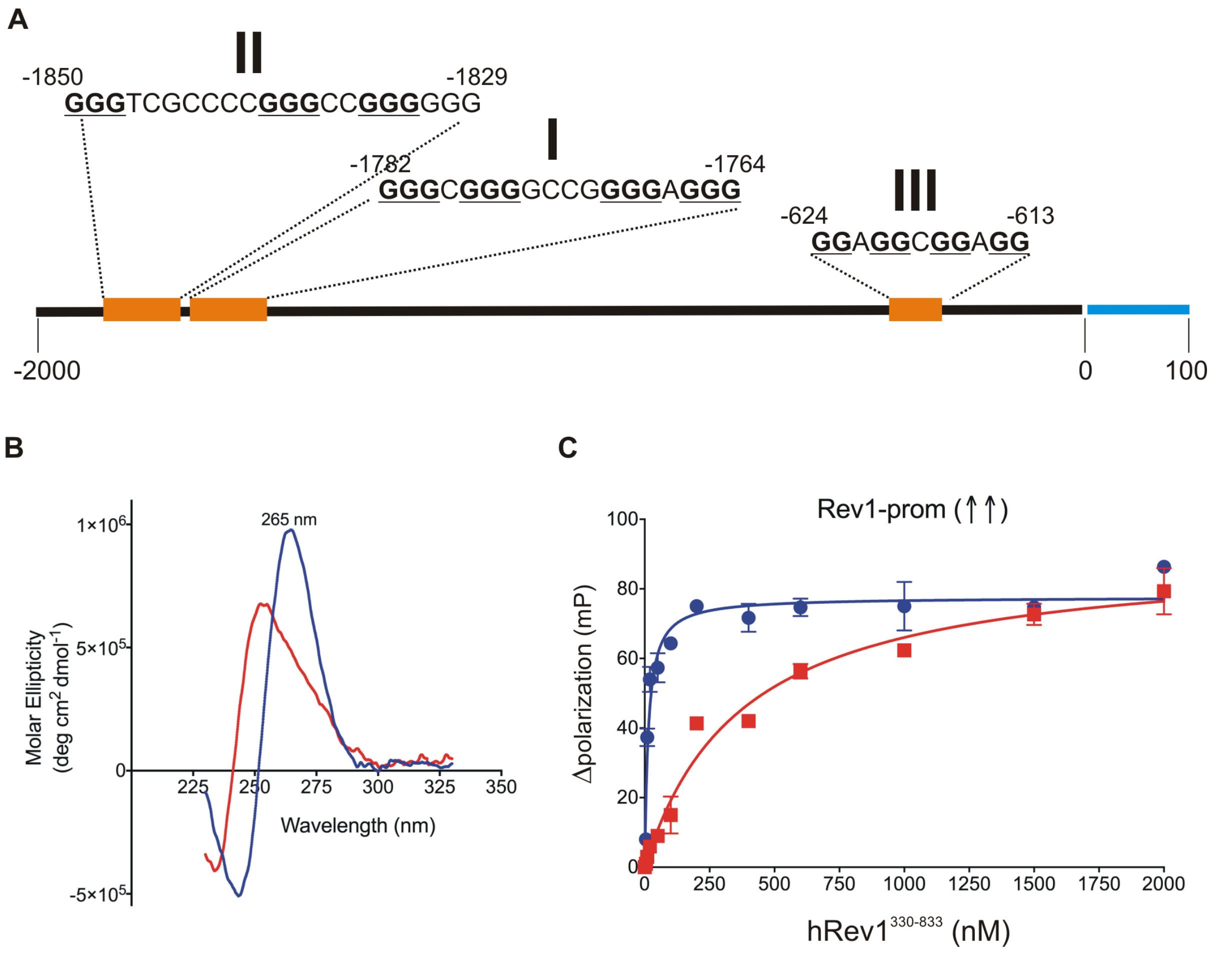

Figure S8. A putative G4-forming sequence was identified at the *REV1* promoter. The human *REV1* gene is located on chromosome 2 (2q11.2; 99490201-99490218 on the negative strand). (**A**) Cartoon schematic showing the region spanning -2000 to +100 bp at the *REV1* gene locus. The transcription start site (marked by position '0') is indicated, as well as the region +100 bp into the coding region (*blue*). DNA sequence was retrieved from the Eukaryotic promoter database and the G4-forming prediction was done by analyzing the sequence using the G4-prediction tool on the QGRS web server. The three top-scoring sequences (based on G-score>20) are marked as I (G-score = 42), II (G-score = 35) and III (G-score = 21) respectively, and the DNA sequences are shown, with the guanine bases involved in putative quadruplex formation marked in bold and underlined. Sequence 'I' was used for designing the Rev1-prom oligonucleotide used in this study. The position of each sequence is marked by the numbers on the 5′- and 3′-end. (**B**) Circular dichroism spectra of the Rev1-prom sequence (**Table 1** in main text) were measured in buffer containing 100 mM KCl (*blue* curve) or 100 mM LiCl (*red* curve). The position of the signature peak at 265 nm for parallel

G4-form is indicated. (**C**) Binding affinity for Rev1-prom G4 (*blue*) and nonG4 (*red*) DNA was measured for the wild-type hRev1$^{330\text{-}833}$ protein as indicated. The measured values are listed in **Table 3** of main text. Reported values represent the mean ± SD (n=3).

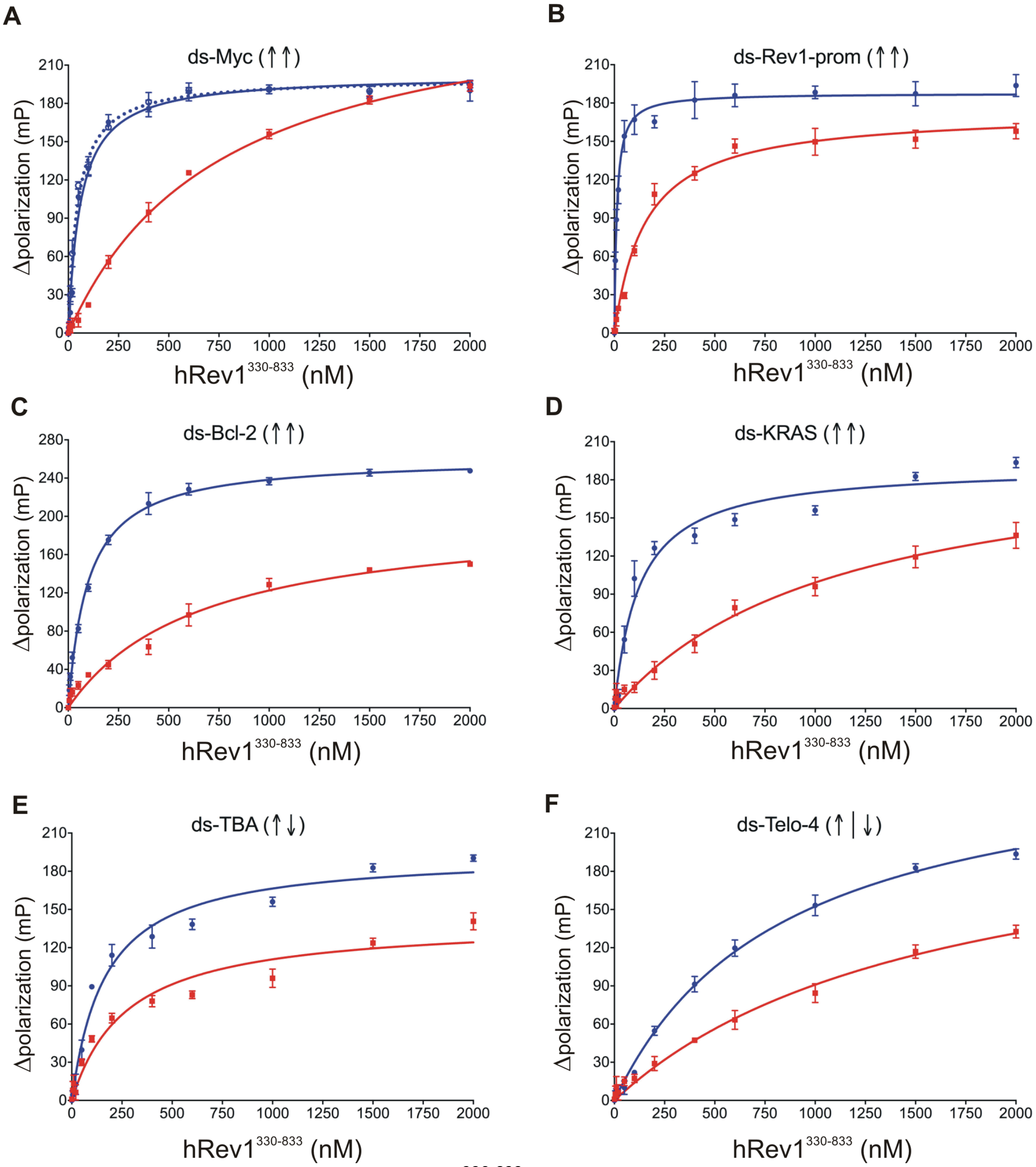

Figure S9. Binding affinity of hRev1$^{330\text{-}833}$ to primer-template G4 DNA substrates. Oligonucleotides forming the different types of G4 DNA folds (described in main text and **Table 1**) were annealed to a common 11-mer primer to obtain primer-template substrates. The hRev1$^{330\text{-}833}$ protein was then titrated into a solution containing each of these ds-G4-DNA (*blue*) or ds-non-G4-DNA (*red*) substrates at 1 nM in a buffer containing 100 mM KCl. The range of concentrations for the protein is indicated on the X-axis. The change in fluorescence polarization at each concentration was measured and plotted as a function of the protein concentration. (**A-F**) Binding curves for hRev1$^{330\text{-}833}$ core protein with the indicated G4 DNA

substrate. In panel **A**, the binding curve for Myc-14/23 is shown as a solid *blue* line (full circles), while that for Myc-2/11 is shown as a dotted *blue* line (open circles). The G4 fold is indicated by the direction of arrows in parentheses for each panel (↑↑ = parallel G4, ↑↓ = anti-parallel G4, ↑|↓ = hybrid G4). Resulting data were fit to a quadratic equation to yield the binding dissociation constants given in **Table S3**. Reported values represent the mean ± SD (n=3).

```
human             HS--NTKINGAHHSTVQGPSSTKSTSSVSTFSKAAPSVPSKPSDCNFISNFYSHSRLHHI 361
chimpanzee        HS--NTKINGAHHSTVQGPSSTKSTSSVSTFSKAAPSVPSKPSDCNFISNFYSHSRLHHI 361
gorilla           HS--NTKINGAHHSTVQGPSSTKSTSSVSTFSKAAPSVPSKPSDCNFISNFYSHSRLHHI 361
rhesus            HS--NTKINGAHHSTVQGPSSTKSTSSVSTLSKAAPSVPSKPSDCNFISNFYSHSRLHHI 395
cattle            HS--NPRVNGAHHSTVQGPSSTKSTSSVPSPSRGRPSGPPRPSDFNFISDFYSRSRLHHI 361
sheep             HS--NPRVNGAHHSTVQGPSSTKSTSSVPSPSRAGPSGPPRPSDCNFISDFYSRSRLHHI 355
pig               HS--NTRVNGAHHSTVQGPSSTRSTSSVPMPSKAAPSVPPRPSDCNFISDFYSRSRLHHI 401
mouse             HS--NTKINGAHHSTVQGPSSTKSTS-VLTLSKVAPSVPSKPSDCNFISDFYSRSRLHHI 359
dog               HS--NTKINGAHHSAVQGPSSTKSTSSVPTLSKAAPLVPSKPSDCNFISDFYSRSRLHHI 361
cat               HS--NTKINGAHHSAVQGPSSTKST-SVPALSKAAPSVPSKPSDCNFISDFYSHSRLHHI 360
opossum           HS--HTKINGAHHSTVQGPSSTKSTSSLPAVSKAASSALSKPSDCNFISDFYSRSRLHHI 363
bat               HT--NTKINGAHHSTVQGPSSTKST-SVPSLSKAAPSVPSRPSDCSFISDFYSHSRLHHI 360
frog              HT--NSKINGAHHSL-PGPSSTSTTA--LSPAKLASLQLPKTADPNFISDFYSHSRLHHI 358
lizard            HS--NIKRNGAHHSTVQGPSSTKSTT---VPSNKVESPLSKPPNCSFISDFYSRSRLHHI 355
zebrafish         HDQTTVRLNGSYHVTSN----------SSTLANSSNQSGKSSAEAGIISEFFSHSRLHHI 391
chicken           HS--NTKINGAHHSTVQGPSSTKSTS-VPTPSKAASLSVSKPSDCSFISDFYSRSRLHHI 366
fruitfly          TN----------------L----------STTSNNSTTARTAADPNFLSEFYKNSRLHHI 231
leishmania        MTAMS-R--------------------------------SVRDQANSLQHFQQHSRLYFI 30
trypanosoma       MSGKK-N--------------------------VSVGRKWACDQVNSVRHFQQHSRLHFI 33
saccharomyces     ------------------------------LNNLEAKRIVACDDPDFLTSYFAHSRLHHL 328
fission_yeast     HNY--------------------------QLLKNPNVRNSTTQNQDFLENFFSSSRLHHL 244
arabidopsis       HSTSDNK--SVHA---NGKNGGK---SIATAAGSSTRRHSTLEDPNFVENYFKNSRLHFI 339
                                                                     : .:: .::  ****.:


human             SMWKCELTEFVNTLQRQ----SNGIFPGREKLKKMKTGRSALVVTDTGDMSVLNSPRHQS 417
chimpanzee        SMWKCELTEFVNTLQRQ----SNGIFPGREKLKKMKTGRSALVVTDTGDMSVLNSPRHQS 417
gorilla           SMWKCELTEFVNTLQRQ----SNGIFPGREKLKKMKTGRSALVVTDTGDMSVLNSPRHQS 417
rhesus            SMWKCELTEFVNTLQRQ----SNGIFPGREKLKKMKTGRSALVVTDTGDMSVLNSPRHQS 451
cattle            SAWKWELTEFVNALQKQ----SSGVFPGREKLKKMKAGRSALVVTDTGNMSVLSSPRHQS 417
sheep             STWKCELTEFVNALQRQ----SSGIFPGREKLKKMKAGRSALVVADTGNMSVLSSPRHQS 411
pig               SMWKCELTEFVNTLQRQ----SSGIFPGREKLKKMKTGRSALVVTDTGNMSVLSSPRHQS 457
mouse             STWKCELTEFVNTLQRQ----SSGIFPGREKLKKVKTGRSSLVVTDTGTMSVLSSPRHQS 415
dog               SMWKCELTEFVNTLQRQ----SSGVFPGREKLKKMKTGRSTLVVTDTGNMSVLSSPRHQS 417
cat               SMWKCELTEFVNTLQRQ----SSGIFPGREKLKKMKTGRSALVVTDTGNMSVLSSPRHQS 416
opossum           SMWKCELTEFVNTLQRQ----SSGVFPGREKLKKMKASRSALIITDAGNMSVLNPSRHQS 419
bat               SMWKCEFTEFVNTLQRQ----SSGIFPGREKLKKMKTGRSALVVTDTGNMSVLSSPRHQS 416
frog              STWKCEFTEFVRGLQTQ----SNKGFPGRERLKKLKPG----------NMPPSTLPKCQN 404
lizard            ATWKSEFTDFVNTLQKQ----NNAIFPGREKLKKLKAGQSILNKADSDYAATLSSTKHQS 411
zebrafish         STWRNEFSEYVNSLQSRRRAAGGAVFSGREKLKKLKANCNSVSHFDPGSLMA-APQVRQS 450
chicken           STWKCELTEFVNSLQRK----NSGVFPGREKLKKWKAGRSAL-KTDTGNVSVASSAKPQS 421
fruitfly          ATLGAGFKQYVCRLRQK---HGTQGFPKRETLKSLANSH---------------HNCLER 273
leishmania        GQWRTKMEDVFR---------------------------EWFIEHPEWNHGTL---EQQR 57
trypanosoma       GQWKTKAHDLFK---------------------------DWFAVHPTWNHGVL--SHQA- 63
saccharomyces     SAWKANLKDKFLNENIH-------------KYT-------------------KITDKDTY 356
fission_yeast     STWKADFKNEIQAMTTA-------SEPVRPIM--------------------KDKSKKSR 277
arabidopsis       GTWRNRYRKRFHGSSNG--------------LK----------WADSGQN--TAEMAKKS 373
                  .       . .
```

```
human          CIMHVDMDCFFVSVGIRNRP------DLKGKPVAVTSNRGTGRA---PLRPGANPQLEWQ 468
chimpanzee     CIMHVDMDCFFVSVGIRNRP------DLKGKPVAVTSNRGTGRA---PLRPGANPQLEWQ 468
gorilla        CIMHVDMDCFFVSVGIRNRP------DLKGKPVAVTSNRGTGRA---PLRPGANPQLEWQ 468
rhesus         CIMHVDMDCFFVSVGIRNRP------DLKGKPVAVTSNRGTGRA---PLRPGANPQLEWQ 502
cattle         CIMHVDMDCFFVSVGIRNRP------DLKGKPVAVTSNRGTGRA---PLRPGANPQLEWQ 468
sheep          CIMHVDMDCFFVSVGIRNRP------DLKGKPVAVTSNRGTGRA---PLRPGANPQLEWQ 462
pig            CIMHVDMDCFFVSVGIRNRP------DLKGKPVAVTSNRGTGRA---PLCPGANPQLEWQ 508
mouse          CVMHVDMDCFFVSVGIRNRP------DLKGKPVAVTSNRGTGTA---PLRPGANPQLEWQ 466
dog            CIMHVDMDCFFVSVGIRNRP------DLKGKPVAVTSNRGTGRA---PLRPGANPQLEWQ 468
cat            CIMHVDMDCFFVSVGIRNRP------DLKGKPVAVTSNRGTGRA---PLRPGANPQLEWQ 467
opossum        CIMHVDMDCFFVSVGIRNRP------DLKGKPVAVTSNRGTGRA---PLRPGANPQLEWQ 470
bat            CIMHVDMDCFFVSVGIRNRP------DLKGKPVAVTSNRGTGRA---PLRPGANPQLEWQ 467
frog           CIIHVDMDCFFVSVAIRNRP------DLKGKPVAVTSNRGAGTT---TTREGANPQLEFQ 455
lizard         CIMHVDMDCFFVSVGIRGRP------DLKGKPVAVTSNRGSGKS---LLRPGANPQLEWQ 462
zebrafish      CVLHVDMDCFFVSVGIRHRP------DLIGKPVAVTSNRGPGRV---AQRPGANPQLEFQ 501
chicken        CIMHVDMDCFFVSVAIRNRP------DLKGKPVAVTSNRGAGKA---PLRPGANPQLEWQ 472
fruitfly       YVMHIDMDCFFVSVGLRTRP------ELRGLPIAVTHSKGGNAATDVPVHPQADRKAELE 327
leishmania     TFVHVDMDAFFCSVQLA-KP---EYAHLKTKPVGIAAGK----------------------  92
trypanosoma    LFVHLDMDAFFCSVVLA-KE---ENAHMREKPVCIAAGK----------------------  98
saccharomyces  IIFHIDFDCFFATVAYLCRSSSFSACDFKRDPIVVCHG---------------------- 394
fission_yeast  FLLHVDFDCFFASVSTRFSH------ELRLKPVAVAHG---------------------- 309
arabidopsis    TIIHIDLDCFFVSVVIKNRL------ELHDKPVAVCHSDNP------------------- 408
                ::*:*:****.:*            ::    *:.*   .


human          YYQNKILKGKAA-----DIPDSSLWENPDSAQANGIDS---VLSRAEIASCYEAR---- 516
chimpanzee     YYQNKILKGKAA-----DIPDSSLWENPDSAQANGIDS---VLSRAEIASCYEARSLKV 520
gorilla        YYQNKILKGKAA-----DIPDSSLWENPDSAQANGIDS---VLSRAEIASCYEARSLKV 520
rhesus         YYQNKILKGKAA-----DIPDSSLWENPDSAQANGIDS---VLSKAEIASCYEAR---- 550
cattle         YYQNKILRGKAA-----EIPDPSTWESQDSAQINGIDS---VLSKAEIASCYEAR---- 516
sheep          YYQNKILRGKAA-----EIPDPSTWESQDSAQINGIDS---VLSKAEIASCYEAR---- 510
pig            YYQNKLLKGRAA-----EIPDSSMWENQDSAQTNGIDY---VLSKAEIASCYEAR---- 556
mouse          YYQNRALRGKAA-----DIPDSSVWENQDSTQTNGIDS---VLSKAEIASCYEAR---- 514
dog            YYQNKILKGKAA-----EIPDSSMWENQDSAQANGIDS---VLSKAEIASCYEAR---- 516
cat            YYQNKILKGKAA-----KIPDSSMWENQDSAQANGIDS---VLSKAEIASCYEAR---- 515
opossum        YYQNKILKGKAAD-RVPDVPDASLLENQDSAQTNGIDS---VLSKAEIASCYEAR---- 522
bat            YYQNKILKGKAA-----DMPDSSLWENQDSAQTNGIDS---VLSKAEIASCYEAR---- 515
frog           YYQNRILKGKAEIPNEQDQP---TSISSDSAQQNGLDLDISHLSMAEIASCYEAR---- 508
lizard         YYQNKLLNGKAAGATELDSPD---DDNPESTQVNGIDLEISVQSMAEIASCYEAR---- 515
zebrafish      YYQNKQKHYRKEKTDG-D-LEMTPSPQDGEVPSNGVHEDLAALSMAEIASCYEAR---- 555
chicken        YYQNKLLNGKAAEIRVPDKLDSWVWEHSDSAHMNGVDCDLTVLSMAEIASCYEAR---- 528
fruitfly       LFAQRFEHHFHDG-------------DKAEKVRSGFDK---KMSLSEIASCYEAR---- 367
leishmania     -------------------------------------------YNSDISSCNYVAR---- 105
trypanosoma    ------------------------------------------GNSDISSS-NYIAR---- 111
saccharomyces  ------------------------------------------TKNSDIASCNYVAR---- 408
fission_yeast  ------------------------------------------IKNSEIASCNYEAR---- 323
arabidopsis    ------------------------------------------KGTAEISSANYPAR---- 422
                                                           ::*:*..* **
```

Figure S10. Alignment of the Rev1 polymerase domain from multiple species. Name of each species is indicated on the left of each sequence as its trivial/common name: human, *Homo sapiens*; chimpanzee, *Pan troglodytes*; gorilla, *Gorilla gorilla gorilla*; rhesus, *Macaca mulatta*; cattle, *Bos taurus*; sheep, *Ovis aries*; pig, *Sus domesticus*; mouse, *Mus musculus*; dog, *Canis familiaris*; cat, *Felis catus*; opossum, *Monodelphis domestica*; bat, *Desmodus rotundus*; frog, *Xenopus tropicalis*; lizard, *Zootoca vivipara*; zebrafish, *Danio rerio*; Chicken, *Gallus gallus* domesticus; fruitfly, *Drosophila melanogaster*; leishmania *Leishmania donovani*; trypanosome, *Trypanosoma cruzi*; saccharomyces, *Saccharomyces cerevisiae*; fission yeast,

Schizosaccharomyces pombe; arabidopsis, *Arabidopsis thaliana*. The top sequence is of the human Rev1 protein, with the domains colored as: *orange*, N-digit (a.a. 344-377); *light grey*, insert 1 (a.a. 379-417); *red*, palm 1 (a.a. 418-425); *blue*, finger (a.a. 427-447 & 505-516); *dark grey*, insert 2 (a.a. 448-504).

REFERENCES

1. Seidman,M.M., Dixon,K., Razzaque,A., Zagursky,R.J. and Berman,M.L. (1985) A shuttle vector plasmid for studying carcinogen-induced point mutations in mammalian cells. *Gene*, **38**, 233–237.
